# The angiopoietin receptor Tie2 is atheroprotective in arterial endothelium

**DOI:** 10.1038/s44161-023-00224-y

**Published:** 2023-03-13

**Authors:** Andrey Anisimov, Shentong Fang, Karthik Amudhala Hemanthakumar, Tiit Örd, Kristof van Avondt, Raphael Chevre, Anu Toropainen, Prosanta Singha, Huda Gilani, Su D. Nguyen, Sinem Karaman, Emilia A. Korhonen, Ralf H. Adams, Hellmut G. Augustin, Katariina Öörni, Oliver Soehnlein, Minna U. Kaikkonen, Kari Alitalo

**Affiliations:** 1 Wihuri Research Institute, Biomedicum Helsinki, University of Helsinki, Helsinki, Finland; 2 Translational Cancer Medicine Program, Biomedicum Helsinki, University of Helsinki, Helsinki, Finland; 3 School of Biopharmacy, China Pharmaceutical University, Nanjing, P. R. China; 4 A.I. Virtanen Institute for Molecular Sciences, University of Eastern Finland, Kuopio, Finland; 5 Institute of Experimental Pathology (ExPat), Center of Molecular Biology of Inflammation (ZMBE), University of Münster, Münster, Germany; 6 Individualized Drug Therapy Research Program, Biomedicum Helsinki, University of Helsinki, Helsinki, Finland; 7 Institute for Neurovascular Cell Biology, University Hospital Bonn, University of Bonn, Bonn, Germany; 8 European Center for Angioscience (ECAS), Medical Faculty Mannheim, Heidelberg University, Mannheim, Germany; 9 Department of Tissue Morphogenesis, Max Planck Institute for Molecular Biomedicine, University of Münster, Münster, Germany; 10 Vascular Oncology and Metastasis, German Cancer Research Center (DKFZ), Heidelberg, Germany; 11 Present Address: Orion Corporation, Orion Pharma, Turku, Finland

**Keywords:** Cardiovascular diseases, Angiogenesis

## Abstract

Leukocytes and resident cells in the arterial wall contribute to atherosclerosis, especially at sites of disturbed blood flow. Expression of endothelial Tie1 receptor tyrosine kinase is enhanced at these sites, and attenuation of its expression reduces atherosclerotic burden and decreases inflammation. However, Tie2 tyrosine kinase function in atherosclerosis is unknown. Here we provide genetic evidence from humans and from an atherosclerotic mouse model to show that TIE2 is associated with protection from coronary artery disease. We show that deletion of *Tie2*, or both *Tie2* and *Tie1*, in the arterial endothelium promotes atherosclerosis by increasing Foxo1 nuclear localization, endothelial adhesion molecule expression and accumulation of immune cells. We also show that Tie2 is expressed in a subset of aortic fibroblasts, and its silencing in these cells increases expression of inflammation-related genes. Our findings indicate that unlike Tie1, the Tie2 receptor functions as the dominant endothelial angiopoietin receptor that protects from atherosclerosis.

## Abstract (web summary)

Anisimov, Fang et al. report that in humans, genetic variants associated with higher TIE2 expression are predicted to confer a reduced risk of CAD; in mice, Tie2 expression in endothelial cells is atheroprotective, and in a subset of aortic fibroblasts, anti-inflammatory.

## Main

Atherosclerosis is characterized by lipid retention and accumulation of macrophages and T cells in the arterial wall, provoking chronic inflammation and accumulation of connective tissue components in atherosclerosis-prone sites in large- and medium-sized arteries^1,2^. Atherosclerosis development is increased by numerous risk factors, including hyperlipidemia, diabetes and hypertension, and is characterized by the appearance and growth of atherosclerotic plaques, mainly at sites of disturbed laminar blood flow^1,3^. Immune cells contribute to the formation of atherosclerotic lesions by creating focal sites of vascular inflammation, which is a critical factor in atherosclerosis progression^4^. Recent clinical trials using anti-inflammatory therapy by blocking the interleukin-1β pathway have led to reduced atherosclerosis^5^. However, the mechanisms of inflammation-induced atherosclerosis remain incompletely understood.

The angiopoietin (Angpt)–Tie signaling pathway is essential for embryonic cardiovascular development and is involved in vascular remodeling during inflammation, tumor angiogenesis and metastasis^6,7^. Unlike many other endothelial cell-specific tyrosine kinase receptors, Tie2 is phosphorylated in the steady-state vasculature, where its activity is controlled by various factors, including the Angpt ligands, the orphan Tie1 receptor, and vascular endothelial protein tyrosine phosphatase^8^. Angpt1, Angpt2 and Angpt4 are highly homologous Tie2 ligands. Angpt1 polypeptides form multimeric complexes and are produced by non-endothelial cells, whereas Angpt2 polypeptides form mainly dimers or low-order multimers and are produced mainly by endothelial cells, in which they form a latent ligand pool that is stored in Weibel–Palade bodies in steady-state conditions^6^.

Disturbed blood flow is associated with local inflammation and early atherosclerotic lesions^1^. Blood flow and laminar shear stress also regulate Tie1 and Tie2 activities^9^. Severe inflammation or tissue damage leads to decreased Tie2 expression, increased Angpt2/Angpt1 ratio, and cleavage of the Tie1 ectodomain, which attenuate endothelial barrier function and sensitize the endothelium to permeability mediators, thus increasing vascular leak^10–13^. As altered levels of Angpt ligands may also decrease homeostatic Tie2 signaling in quiescent vasculature and as endothelial Tie2 signaling can affect leukocyte extravasation^6,14,15^, Tie2 could be involved in the development of atherosclerosis.

Previous work has shown that diminished Tie1 expression in apolipoprotein E-deficient (*ApoE*^−/−^) mice decreases atherosclerosis development, in part by reducing vascular inflammation^16^, indicating that Tie1 has a pro-inflammatory function in atherosclerosis. Prolonged systemic expression of Angpt2 induces robust Tie2 phosphorylation^11,12,17^ and is atheroprotective^18^. Although administration of Angpt2 blocking antibodies did not affect pre-existing aortic lesions in *ApoE*^−/−^ mice, it decreased the development of fatty streaks and early plaque formation^19^. Angpt1, on the other hand, aggravated atherosclerosis by promoting inflammatory responses^20,21^. Because of these contrasting results concerning the Tie2 ligands, we examined whether Tie2 is an essential factor during atherosclerosis formation.

In this Article, we provide evidence that variation of arterial TIE2 expression is associated with the development of coronary atherosclerosis in humans. We then use genetic deletion of *Tie2* alone or in combination with *Tie1* in arterial endothelial cells (AECs) in mice to explore Tie2 function in atherosclerosis.

## Results

### Variation of *TIE2* in human coronary atherosclerosis

**Extended Data Fig. 1:**
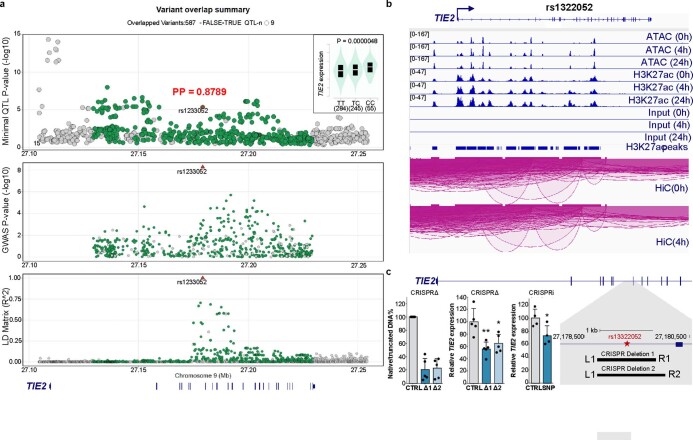
GWAS identify TIE2 (TEK) as a risk locus for CAD. **a**. A LocusZoom plot with eQTL association in GTEX v8 Tibial Artery (top) and CAD GWAS association (middle) and LD matrix (bottom) generated using ezQTL tool. The red triangle indicates the rs1322052 SNP which demonstrates strong colocalization between eQTL and GWAS signals within the LD block (HyPrColoc posterior probability, PP: 0.8789). The insert in top figure indicates the direction of eQTL association with the risk variant T being associated with lower TIE2 expression. **b**. IGV^80^ genome browser screenshots of the TIE2 locus that demonstrates ATAC-Seq, H3K27ac ChIP-Seq and Hi-C data from teloHAECs treated with 0–24 TNFα stimulus^65^. The rs1322052 localizes to cis-regulatory enhancer element that is TNFα-responsive and loops to the TIE2 promoter. **c**. CRISPR deletion (CRISPRΔ) and CRISPR inhibition (CRISPRi) experiments were used to study the effect of rs1322052 variant site on *TIE2* expression in teloHAECs. Two pairs of gRNAs were used to make the deletion of 945 bps and 1120 bps centered around rs1322052 variant (schematic). Quantification of DNA band intensities on the gel image demonstrate ~80% deletion of the intronic rs1322052 variant (bar plot on the left). The deletion translated to 35–44% reduction in the expression of *TIE2* (*TEK*) gene (bar plot in the middle). CRISPRi-mediated inhibition of the variants site led to ~30% repression of *TIE2* expression (bar plot in the right). Data represented as means ± s.d. (for CRISPRΔ, *n* = 5 independent samples per group, one-way ANOVA (*P* = 0.0024) with Dunnett’s post hoc test (*P < 0.05 and **P < 0.01); for CRISPRi, *n* = 3 independent samples per group, two-tailed *t*-test, unpaired *P* = 0.0379).

Previously, rare genetic variants in *TEK* gene (*encoding* *TIE2*) have been shown to contribute to congenital heart disease^22^. However, because the large sample sizes in genome-wide association studies (GWAS) improve candidate gene discovery for common diseases such as coronary artery disease (CAD), we took advantage of a meta-analysis of multiple GWAS^23–27^ from approximately 1.5 million individuals provided by the Cardiovascular Disease Knowledge Portal (broadcvdi.org, 2021 11/30) to examine the association of variants of the *TIE2* gene with cardiovascular disease and related traits. Notably, this analysis revealed that the *TIE2* gene is significantly associated with CAD (*P* = 5.238 × 10^−9^; Extended Data Fig. 1a). Interestingly, the top associated intronic variant rs1322052 was also significantly associated with the expression of *TIE2* in tibial arteries in the Genotype-Tissue Expression (GTEx) v8 database^28^, suggesting a functional effect of this single-nucleotide polymorphism (SNP) (Extended Data Fig. 1a). Importantly, the C allele conferred reduced risk of CAD (Effect size: -0.0278) and was associated with increased expression of *TIE2*. HyPrColoc analysis further demonstrated significant colocalization (posterior probability of 0.8789) between the GWAS and expression quantitative trait loci (eQTL) signals, supporting sharing of the same causal variant rs1322052. Additional analysis of the chromatin characteristics of the locus in hTERT-immortalized human aortic endothelial cells (teloHAECs) suggested that the risk variant is located within a tumor necrosis factor-α (TNF-α)-responsive enhancer element, which is connected through chromatin interaction to the *TIE2* promoter (Extended Data Fig. 1b). CRISPR-mediated deletion and inhibition of the enhancer led to a significant decrease in *TIE2* mRNA expression, providing additional evidence of its functional effect (Extended Data Fig. 1c). Taken together, this indicates that a genetic variant affecting *TIE2* expression, especially in the arteries, could be associated with the development of human coronary atherosclerosis.

### *Tie2* deletion leads to increased atherosclerosis in mice

**Extended Data Fig. 2:**
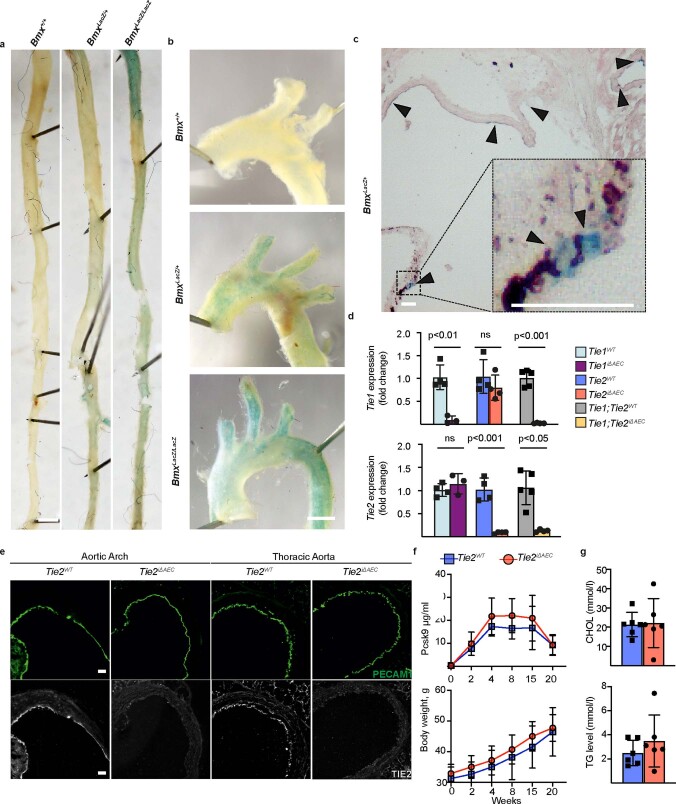
Expression of *Bmx* gene and confirmation of *Tie1* and *Tie2* gene deletions in the aorta. **a**. Representative images of β-gal staining in the thoracic aorta, **b**. aortic arch and **c**. aortic root from WT (*Bmx*^*+/+*^) mice and in mice expressing the *LacZ* gene targeted into the *Bmx* locus. Scale bar, 1 mm (A-B) and 50 μm (C). **d**. Fold differences of *Tie1* and *Tie2* transcripts in the *Tie1*^*iΔAEC*^ (*n* = 3 independent mice per group, Tie1: *P* = 0.0025), *Tie2*^*iΔAEC*^ (*n* = 4 independent mice per group, Tie2: *P* = 0.0003), and *Tie1;Tie2*^*iΔAEC*^ mice (*n* = 5 independent *Tie1;Tie2*^*WT*^ mice and *n* = 4 independent *Tie1;Tie2*^*iΔAEC*^ mice, Tie1: *P* = 0.0003,Tie2: *P* = 0.0015) in comparison with their littermate controls. Statistical significance was determined using Student’s *t*-test (two tailed, unpaired). **e**. Immunofluorescence of Tie2 and PECAM1 in the aortic root, aortic arch and thoracic aorta of the *Tie2*^*iΔAEC*^ and *Tie2*^*WT*^ mice (*n* = 3 independent mice per group). Scale bar, 50 μm. **f**. Pcsk9-D377Y concentration in serum samples and body weight measurements at the indicated time points in the *Tie2*^*iΔAEC*^ and *Tie2*^*WT*^ mice (*n* = 6 independent mice per group)**. g**. Quantification of cholesterol (CHOL) and triglyceride (TG) concentrations in serum samples from the Tie2-deleted mice (*n* = 6 independent mice per group) 20 weeks after Pcsk9 and Western diet. Values show mean ± s.d. Statistical significance was determined using Student’s *t*-test (two tailed, unpaired). Source data

Motivated by the GWAS finding that a higher expression of TIE2 could protect from coronary atherosclerosis, we investigated the effect of *Tie2* deletion in the arteries in mice subjected to hypercholesterolemia by systemic expression of an activated form of proprotein convertase subtilisin/kexin type 9 (Pcsk9-D377Y) and high-fat feeding (Western diet). To delete the *Tie2* gene in aortic AECs, we crossed the mice carrying *Tie2*^*fl/fl*^ alleles (designated here as *Tie2*^*WT*^) with *BmxCre*^*ERT2*^ deletor mice to obtain *BmxCre*^*ERT2*^;*Tie2*^*fl/fl*^ mice (designated here as *Tie2*^*iΔAEC*^). We confirmed *Bmx* expression in the aortic endothelium (aortic arch and descending part of thoracic aorta) by β-galactosidase staining using mice, in which the *LacZ* gene was used to replace one copy of the *Bmx* gene (*Bmx*^*LacZ*^ mice) (Extended Data Fig. 2a–c). After tamoxifen treatment, *Tie2* deletion in AECs (*Tie2*^*iΔAEC*^) was verified by real-time quantitative PCR (qPCR) analysis of aortic RNA and by immunofluorescence staining of aortic sections (Extended Data Fig. 2d, e).

**Fig. 1:**
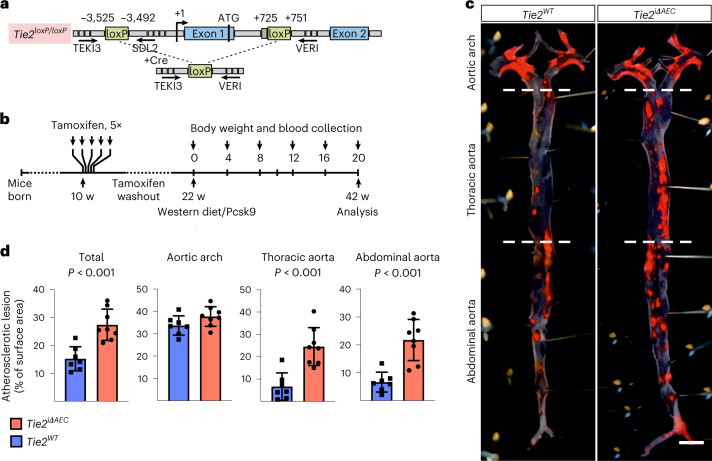
Arterial *Tie2* deletion promotes atheroma formation. **a**, Detailed schematic of the gene deletions. Numbers indicate nucleotide positions of the loxP sites relative to the transcription start site and names of the genotyping primers (Supplementary Table 1). **b**, Outline and time schedule (in weeks, w) of the experimental setup. **c**, Control and *Tie2*-deleted aortas stained with Sudan IV (*n* = 7 independent *Tie2*^*WT*^ and *n* = 8 independent *Tie2*^*iΔAEC*^ mice; scale bar, 2 mm). **d**, Quantification of aortic lesion areas (total, *P* = 0.0004; aortic arch, *P* = 0.099; thoracic aorta, *P* = 0.0005; abdominal aorta, *P* = 0.0003). Values show mean ± s.d. Statistical significance was determined using Student’s *t*-test (two-tailed, unpaired). Source data

After a tamoxifen washout period of 12 weeks, the mice were injected with an adeno-associated virus (AAV) vector encoding Pcsk9-D377Y, which decreases low-density lipoprotein (LDL) receptor expression in the liver. The mice were then subjected to a high-fat Western diet for 20 weeks, during which Pcsk9 serum concentration and body weight were monitored (Fig. 1a, b and Extended Data Fig. 2f). No differences in the cholesterol or triglyceride concentration were observed between the *Tie2*^*WT*^ and *Tie2*^*iΔAEC*^ mice (Extended Data Fig. 2g). We analyzed the formation of atherosclerotic plaques in the atheroprone aortic arch and atheroresistant thoracic and abdominal parts of the aorta by Sudan IV staining. *Tie2* deletion was associated with an increased size and number of the atherosclerotic plaques, especially in the thoracic and abdominal parts of the aorta (Fig. 1c, d). These data suggested that Tie2 function in the arterial endothelium protects mice from atherosclerosis.

### Inflammatory cells are increased in the *Tie2*-deleted aortas

**Fig. 2:**
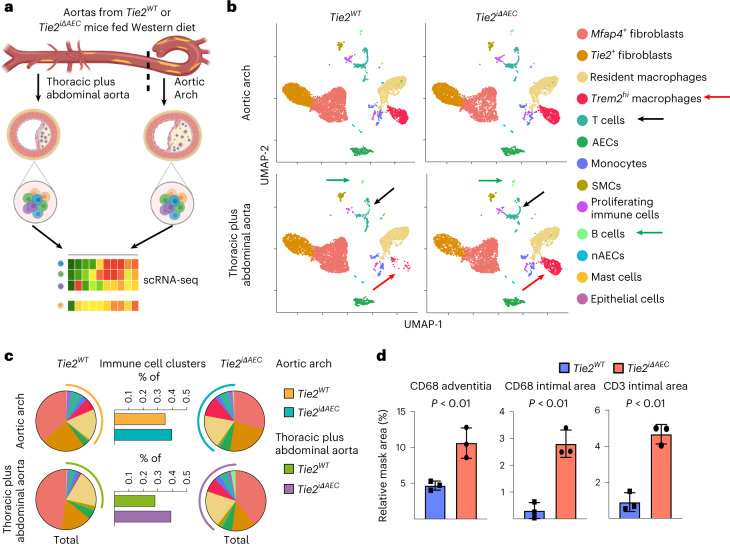
scRNA-seq analysis of cells in the atheromatous aortas. **a**, Schematic of the analysis. **b**, UMAP plots of aligned gene expression data from aortic cells isolated and pooled from 2–3 individual *Tie2*^*WT*^ (*n* = 7,903 in the aortic arch, *n* = 6,310 in the thoracic plus abdominal aorta isolated and pooled from 2–3 mice) and *Tie2*^*iΔAEC*^ (*n* = 6,578 and *n* = 6,178, respectively) mice after 20 weeks of Western diet, partitioned into 13 distinct clusters. *Trem2*^*hi*^ macrophage (red arrow), T cell (black arrow) and B cell (green arrow) clusters showed the most obvious changes after endothelial cell *Tie2* deletion. **c**, Proportions of immune cell clusters in the analyzed aortic regions. **d**, Quantification of CD68 macrophages (adventitia, *P* = 0.0096; intimal area, *P* = 0.0019) and CD3e T cells (*P* = 0.0010) in the atherosclerotic arotas (*n* = 3 independent mice per group). Values show mean ± s.d. Statistical significance was determined using Student’s *t*-test (two-tailed, unpaired). Source data

**Extended Data Fig. 3:**
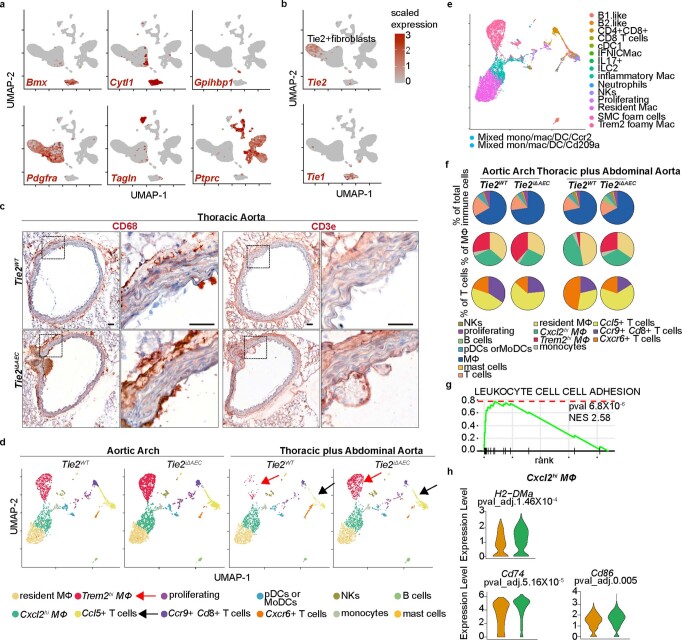
*Tie2*^*iΔAEC*^ is associated with alteration of T cell and macrophage subsets during atherosclerosis. **a**. Feature plot showing the expression of Bmx (AECs), Cytl1 (AECs), Gpihbp1 (nAECs), Pdgfra (fibroblasts), Tagln (SMCs) and Ptprc (immune cells). **b**. Tie1 and Tie2 expression shown in the feature plot. Note that unlike Tie2, Tie1 was not expressed in the Tie2+ FB cluster. **c**. Representative images of immunohistochemical (chromogenic) staining of CD68 macrophages and CD3e T cells in the atherosclerotic aortas (*n* = 3 independent mice per group). Scale bar, 50 μm. **d**. UMAP plots of (12) immune cell clusters from the atheromatous aortic arch and thoracic plus abdominal aortas of the *Tie2*^*WT*^ and *Tie2*^*iΔAEC*^ mice. **e**. UMAP plots of immune cell annotation referenced to the published datasets using SingleR. **f**. Pie plots show the relative proportions of the main immune cell types (macrophage/monocyte, T cells, DCs, B cells, NK and mast cells) (**upper**), macrophages (**middle**), and T cells (**lower**). **g**. Gene enrichment analysis showing pathways that are related the upregulated genes in the Cxcl2^hi^ macrophages in the *Tie2*^*iΔAEC*^ mice. *P* value adjustment is performed using a Benjamini-Hochberg (BH)-procedure (Multiple comparison test) as indicated in the fgsea package. **h**. Violin plots showing the expression of MHC II CLASS gene, CD74 and APC costimulatory molecule CD86 in the Cxcl2^hi^ macrophages. Statistical significance was determined using the Wilcoxon rank-sum test. *P* value adjustment is performed using Bonferroni correction based on the total number of genes in the dataset as indicated in the Seurat package. Source data

To mechanistically link the arterial endothelial *Tie2* deletion to atherosclerosis progression, we analyzed cell type-specific gene expression profiles using single-cell RNA sequencing (scRNA-seq) in the atheroprone aortic arch and the atheroresistant descending thoracic and abdominal aortas from the *Tie2*^*WT*^ and *Tie2*^*iΔAEC*^ mice that were treated with AAV-Pcsk9 and fed a Western diet for 20 weeks (Fig. 2a). The major cell types identified in the atheromatous aortas were *Bmx*-expressing aortic luminal cells (AECs; expressing *Cytl1*) and non-AECs (expressing *Gpihbp1*), fibroblasts (expressing *Pdgfra, Serpinf1* and *Clec3b*), immune cells and smooth muscle cells (SMCs; expressing *Tagln* and *Acta2*), in agreement with previous reports on aortic cells in atherosclerosis^29–31^ (Fig. 2b, Extended Data Fig. 3a and Supplementary Data 1). The fibroblasts were partitioned into two major clusters, one of which, surprisingly, expressed *Tie2* (*Tie2*^+^ fibroblasts), but not *Tie1* (Extended Data Fig. 3b).

Interestingly, in the atheromatous aortas of the *Tie2*^*iΔAEC*^ mice, the proportion of immune cells (*Trem2*^*hi*^ macrophages plus T and B cells) was higher than in the atheromatous aortas of *Tie2*^*WT*^ mice; this effect of *Tie2* deletion was especially prominent in the thoracic and abdominal aorta (Fig. 2b, c). The increase of CD68^+^ macrophages and CD3e^+^ T cells after *Tie2* deletion in the thoracic aorta was confirmed by chromogenic immunostaining (Fig. 2d and Extended Data Fig. 3c). These results indicated that *Tie2* deletion in AECs leads to increased immune cell infiltration, facilitating atherosclerosis development.

To further understand the inflammatory changes in the *Tie2*-deleted atheromatous aortas, we subset the leukocyte clusters and re-clustered them. We identified four macrophage or monocyte clusters (*Lyve1*-expressing resident macrophages, *Ccr2* and *Lyz1*-expressing monocytes, *Trem2*-expressing *Trem2*^*hi*^ macrophages and *Cxcl2/Ccl2*-expressing *Cxcl2*^*hi*^ macrophages), three T cell clusters (*Ccl5*^+^ T cells, *Ccr9/Cd8*^+^ T cells and *Cxcr6*^+^ T cells), one B cell cluster expressing *Cd79b*, one *Cd209a*-expressing dendritic cell cluster, one *Itgal*-expressing natural killer cell cluster, one mast cell cluster expressing *Kit* and one *Top2a*-expressing proliferating immune cell cluster (Extended Data Fig. 3d and Supplementary Data 2). Our analysis indicated that *Trem2*^*hi*^ and *Cxcl2*^*hi*^ macrophages correspond to the Trem2 foamy and inflammatory macrophages, respectively, in the recent meta-analysis published by Zernecke et al.^32^ (Extended Data Fig. 3e). In this report, the Trem2 foamy macrophages showed low expression of inflammatory genes, whereas the inflammatory macrophages that were enriched in the atherosclerotic aortas in our study had a strong pro-inflammatory profile^32^. Ccr9/Cd8^+^ T cells and Ccl5^+^ T cells in our samples resembled the CD4^+^CD8^+^ and CD8^+^ cells in the report by Zernecke et al., whereas Cxcr6^+^ T cells resembled the ILC2 and IL-17 T cells, respectively, and our B cell lineage comprised both the B1-like and B2-like cells in the report by Zernecke et al.^32^

Although the distribution of these seven immune cell populations did not change significantly after *Tie2* deletion, the atheromatous thoracic and abdominal aortas contained fewer *Trem2*^*hi*^ macrophages in the macrophage and monocyte cluster than in the aortic arch (3.3% versus 27.8%; Extended Data Fig. 3f). The *Trem2*^*hi*^ macrophages were increased concomitantly with the increase in atherosclerotic plaques in the thoracic and abdominal aortas of the *Tie2*-deleted mice (Extended Data Fig. 3f). Interestingly, we found that genes involved in leukocyte cell–cell interactions, especially major histocompatibility complex (MHC) class II gene, CD74 and the APC costimulatory molecule CD86, were significantly upregulated in the *Cxcl2*^*hi*^ macrophages in the atherosclerotic thoracic and abdominal aortas of the *Tie2*^*iΔAEC*^ mice (Extended Data Fig. 3g, h and Supplementary Data 2).

*Ccl5*^+^ T cells were more abundant in the aortic arch than in the thoracic and abdominal aorta in the atherosclerotic *Tie2*^*WT*^ mice (45.8 versus 31.4%), and they were further enriched in the atherosclerotic *Tie2*^*iΔAEC*^ aortas, especially in the thoracic and abdominal parts (Extended Data Fig. 3f). *Ccl5*^+^ T cells are known to activate macrophages and promote plaque vulnerability via CCL5–CCR5 signaling^33^. Thus, they may contribute to the increased atherosclerosis in the *Tie2*-deleted mice. The increased levels of *Trem2*^*hi*^ macrophages and *Ccl5*^+^ T cells may, in part, explain the increased atherosclerosis progression in the *Tie2*-deleted mice.

### Immune cell infiltration during the onset of atherosclerosis

**Fig. 3:**
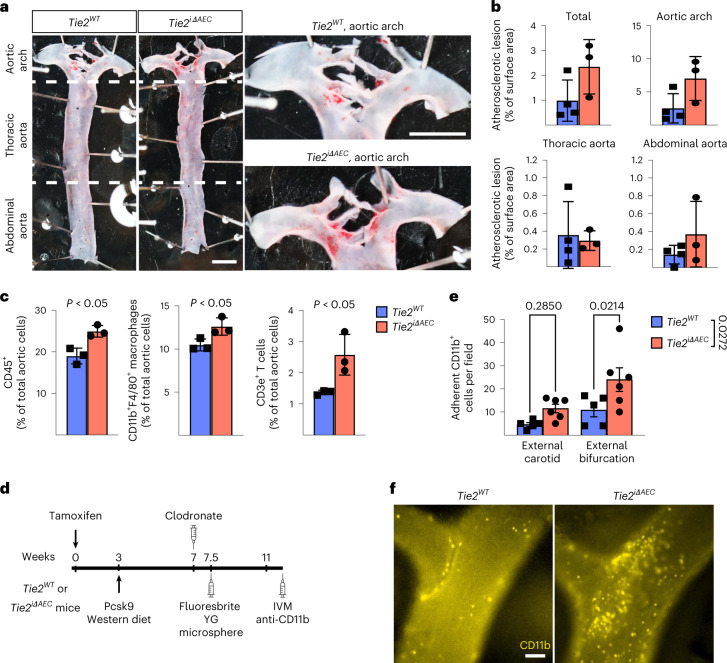
*Tie2* deletion increases inflammatory cells in atherosclerotic lesions. **a**, Representative images of aortas stained with Sudan IV from the *Tie2*^*WT*^ and *Tie2*^*iΔAEC*^ (*n* = 4 independent *Tie2*^*WT*^ and *n* = 3 independent *Tie2*^*iΔAEC*^ mice) mice after 4 weeks of Western diet (scale bar, 2 mm). **b**, Quantification of aortic lesion areas. Values show mean ± s.d. **c**, Quantification of CD45^+^ immune cells (*P* = 0.012), CD11b^+^F4/80^+^ macrophages (*P* = 0.038) and CD3e^+^ T cells (*P* = 0.034) in the aortas (*n* = 3 independent mice per group) using flow cytometry. Values show mean ± s.d. Statistical significance was determined using Student’s *t*-test (two-tailed, unpaired). **d**, Outline and time schedule (in weeks) of the experimental setup in the intravital microscopy analysis. **e**,**f**, Images of CD11b^+^ myeloid cells adherent to the carotid artery wall in the bifurcation area (**f**). Scale bar, 100 µm. Quantification of CD11b^+^ myeloid cells in the external carotid artery and its bifurcation (*n* = 5 independent *Tie2*^*WT*^ and *n* = 6 *Tie2*^*iΔAEC*^ mice) (**e**). Each data point represents one mouse. Values show mean ± s.d. Statistical significance was determined using two-way ANOVA with Sidak post hoc test. Source data

**Extended Data Fig. 4:**
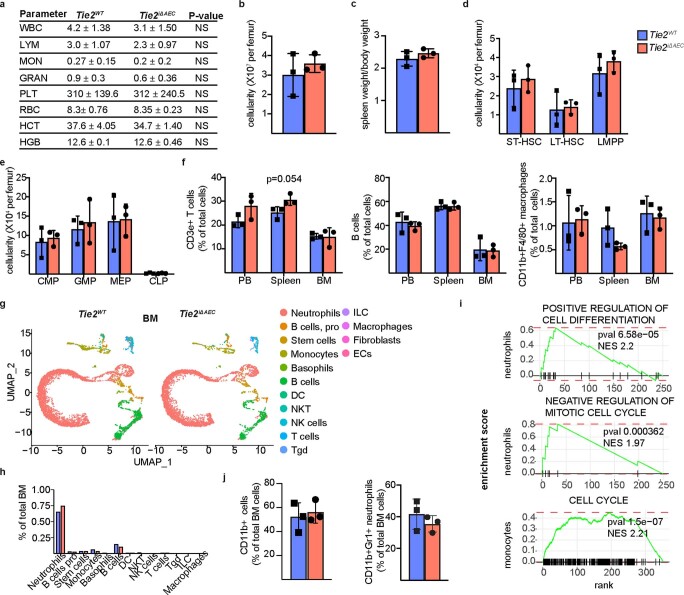
No difference in bone marrow leukocyte production between *Tie2*^*WT*^ and *Tie2*^*iδAEC*^ mice. **a**. Peripheral blood cell counts indices in the *Tie2*^*WT*^ and *Tie2*^*iΔAEC*^ mice four weeks after Western diet. **b-c**. Quantification of BM cellularity and spleen weight/bodyweight in the *Tie2*^*WT*^ and *Tie2*^*iΔAEC*^ mice (*n* = 3 independent mice per group). **d-e**. Quantification of BM stem and progenitor cell numbers in the *Tie2*^*WT*^ and *Tie2*^*iΔAEC*^ mice (*n* = 3 independent mice per group). **f**. Quantification of CD3e+ T cells, B220 + B cells and CD11b + F4/80+ macrophages in the BM from the *Tie2*^*WT*^ and *Tie2*^*iΔAEC*^ mice (*n* = 3 independent mice per group). **g**. UMAP plots of (15) immune cell clusters from red blood cell-depleted total bone marrow of the *Tie2*^*WT*^ and *Tie2*^*iΔAEC*^ mice four weeks after Western diet with Immgen annotation. **h**. Quantification of immune cell clusters from the experiment. Values show mean ± s.d. Statistical significance was determined using Student’s *t*-test (two tailed, unpaired). **i**. Gene enrichment analysis showing pathways that are related the upregulated genes in the BM neutrophils and monocytes in the Tie2^iΔAEC^ mice. *P* value adjustment is performed using a Benjamini-Hochberg (BH)-procedure (Multiple comparison test) as indicated in the fgsea package. **j**. Quantification of CD11b + cells and CD11b + Gr1+ neutrophils in the BM from the *Tie2*^*WT*^ and *Tie2*^*iΔAEC*^ mice (*n* = 3 per group). Values show mean ± s.d. Statistical significance was determined using Student’s *t*-test (two tailed, unpaired). Source data

To evaluate the possible effect of *Tie2* deletion from AECs on the egress of leukocytes from the bone marrow or their recruitment to the aorta, we analyzed the *Tie2*^*WT*^ and *Tie2*^*iΔAEC*^ mice after Pcsk9-D377Y overexpression and Western diet for 4 weeks. We found that during this time period, *Tie2* deletion caused only a trend (*P* = 0.08) of increased plaque formation in the aortic arch, but not in the thoracic or abdominal aorta (Fig. 3a, b). Peripheral white blood cell composition, total bone marrow cellularity, spleen size, bone marrow hematopoietic stem and progenitor cells, and bone marrow lineage composition were similar in the *Tie2*^*WT*^ and *Tie2*^*iΔAEC*^ mice, suggesting that bone marrow leukocyte production and egress from bone marrow, or their mobilization, was not significantly altered in the *Tie2*^*iΔAEC*^ mice during the onset of atherosclerosis (Extended Data Fig. 4a–f). This was further confirmed by scRNA-seq analysis of red blood cell-depleted total bone marrow from the *Tie2*^*WT*^ and *Tie2*^*iΔAEC*^ mice and subsequent annotation of the cell types using SingleR and Immunological Genome Project (ImmGen) datasets^34^, which did not indicate differences in the bone marrow monocyte or T cell profiles (Extended Data Fig. 4g, h). We found a significant upregulation of transcripts related to positive regulation of cell differentiation and negative regulation of mitotic cell cycle in bone marrow neutrophils from the *Tie2*^*iΔAEC*^ mice (Extended Data Fig. 4i and Supplementary Data 3). In contrast, genes related to cell cycle were upregulated in monocytes from the *Tie2*^*iΔAEC*^ bone marrow. However, there was no significant change in total neutrophil or monocyte numbers in the bone marrow (Extended Data Fig. 4j).

**Extended Data Fig. 5:**
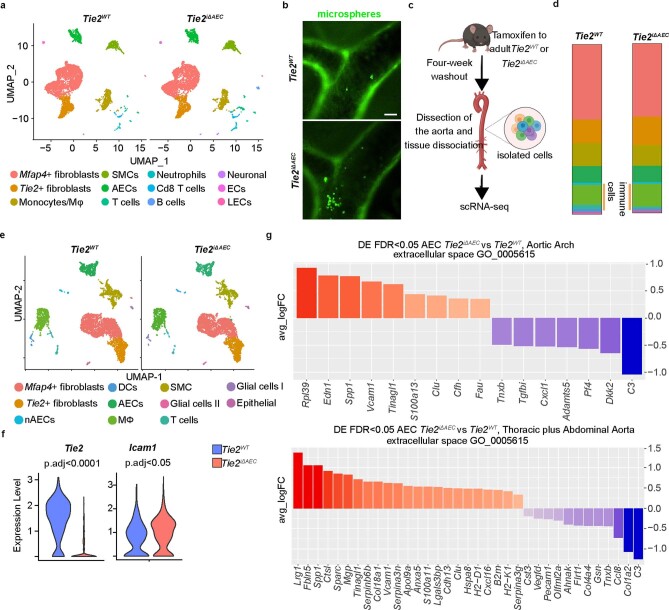
scRNA analysis of *Tie2*^*WT*^ and *Tie2*^*iΔAEC*^ mice on normal diet or after 4 weeks of Western diet. **a**. UMAP plots of total aortic cells from adult *Tie2*^*WT*^ and *Tie2*^*iΔAEC*^ mice after 4 weeks of Western diet. **b**. Representative images of cells labelled with fluorescent microbeads in the carotid arteries of the *Tie2*^*WT*^ and *Tie2*^*iΔAEC*^ mice (*n* = 5 *Tie2*^*WT*^ and *n* = 6 *Tie2*^*iΔAEC*^). Scale bar, 100 µm. **c**. Schematic of the experimental workflow. **d**. Proportions of the distinct clusters and total immune cells among the total aortic cells. **e**. UMAP plots of total aortic cells (*n* = 5936 and *n* = 5615, respectively, that partition into 11 distinct clusters) from adult *Tie2*^*WT*^ and *Tie2*^*iΔAEC*^ mice on normal diet, four weeks after *Tie2* deletion. **f**. *Tie2* (p.adj=2.14×10^-99^) and *Icam1* (p.adj=0.0258) expression in aortic AECs shown in the violin plot. Statistical significance was determined using a Wilcoxon rank-sum test. *P* value adjustment was performed using Bonferroni correction based on the total number of genes in the dataset as indicated in the Seurat package. **g**. Gene ontology annotation analysis of the differentially expressed genes (DEGs) in the AECs in the *Tie2*^*iΔAEC*^ aortas. *P* value adjustment is performed using a Benjamini-Hochberg (BH)-procedure (Multiple comparison test). Source data

Interestingly, total CD45^+^ leukocytes, CD3e^+^ T cells and CD11b^+^F4/80^+^ macrophages were more numerous in the *Tie2*^*iΔAEC*^ aortas than in the *Tie2*^*WT*^ aortas, suggesting increased leukocyte recruitment and adhesion to the aortic wall during the early atherosclerotic events (Fig. 3c). In flow cytometry, only a trend of increase (*P* = 0.054) of splenic T cells, but not monocytes, was observed in the *Tie2*^*iΔAEC*^ mice (Extended Data Fig. 4f). scRNA-seq analysis of the aortas showed an increase in total leukocytes (*Tie2*^*iΔAEC*^ 18.1% versus *Tie2*^*WT*^ 14.8%), especially T cells (*Tie2*^*iΔAEC*^ 4.79% versus *Tie2*^*WT*^ 2.16%), in agreement with the flow cytometric analysis (Extended Data Fig. 5a). Total leukocyte and T cell clusters in the *Tie2*^*iΔAEC*^ and *Tie2*^*WT*^ aortas on normal diet did not differ significantly in the scRNA-seq analysis (Extended Data Fig. 5c–e).

To study if the endothelial inflammation and increased in leukocyte counts in the aortic lesions in *Tie2*^*iΔAEC*^ mice are associated with increased leukocyte adhesion to the aortic luminal wall, we performed intravital microscopy of leukocyte adhesion to the external carotid artery at its bifurcation in the hypercholesterolemic *Tie2*^*WT*^ and *Tie2*^*iΔAEC*^ mice (Fig. 3d). We observed a significant increase in the adherence of fluorescently labeled myeloid cells to the carotid artery, especially in areas flanking its bifurcation in the *Tie2*^*iΔAEC*^ versus *Tie2*^*WT*^ mice (Fig. 3e, f). We also found that some of the microspheres that were injected intravenously 4 weeks before intravital microscopy were associated with the carotid arterial wall in the bifurcation-flanking areas in the *Tie2*^*iΔAEC*^ mice (Fig. 3d and Extended Data Fig. 5b).

**Extended Data Fig. 6:**
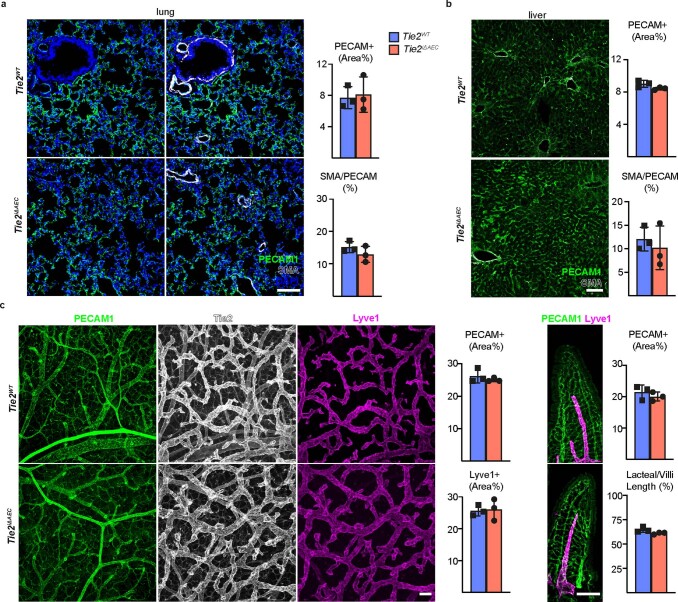
No obvious defects in other vascular beds in the *Tie2*^*iΔAEC*^ mice four weeks after Western diet. **a**. Representative immunofluorescent images of lung sections stained for PECAM1 (green), DAPI (blue), and SMA (white). Quantification of PECAM1 and SMA/PECAM1 ratios (*n* = 3 independent mice per group). **b**. Representative immunofluorescent images of liver sections and their quantification (*n* = 3 independent mice per group). **c**. Representative immunofluorescent images of intestine stained for PECAM1 (green), Tie2 (white), and Lyve1 (red). Quantifications of PECAM1, Lyve1 and lacteal/villus length ratio (*n* = 3 independent mice per group). Values show mean ± s.d. Scale bar, 100 μm. Source data

To ascertain the lack of major unspecific changes in other vascular beds, we confirmed that there were no significant differences in vessels in the lungs, liver or intestine between the *Tie2*^*iΔAEC*^ and *Tie2*^*WT*^ mice 4 weeks after treatment with Pcsk9-D377Y overexpression and Western diet (Extended Data Fig. 6a–c). Collectively, these data show that *Tie2* deletion from AECs facilitates the development of atherosclerosis by increasing immune cell recruitment, without altering bone marrow leukocyte egress or mobilization.

### *Tie2* deletion promotes inflammatory phenotype in AECs

**Fig. 4:**
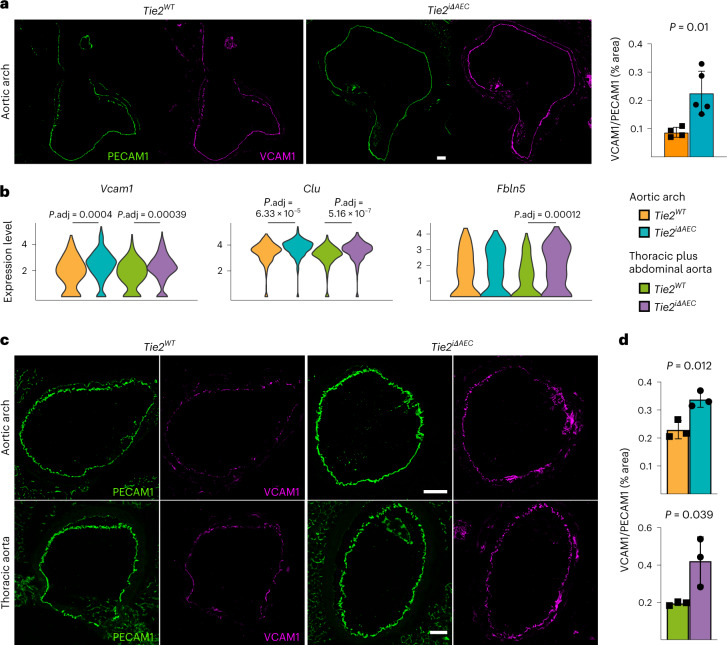
Expression of leukocyte adhesion receptors and transcripts associated with lymphocyte activation in the *Tie2*^*WT*^ and *Tie2*^*iΔAEc*^ mice. **a**, Representative immunofluorescence images of aortic arch from the *Tie2*^*WT*^ and *Tie2*^*iΔAEC*^ mice 8 weeks after Western diet and Pcsk9 overexpression stained for platelet and endothelial cell adhesion molecule 1 (PECAM1; green) and VCAM1 (red) (left). Quantification of the VCAM1/PECAM1 ratio (*n* = 4 independent *Tie2*^*WT*^ mice and *n* = 5 independent *Tie2*^*iΔAEC*^ mice) (right). Scale bar, 100 μm. Values show mean ± s.d. Statistical significance was determined using Student’s *t*-test (two-tailed, unpaired)*.* **b**, Violin plots of *Vcam1*, *Clu* and *Fbln5* expression in AECs. Note that after *Tie2* deletion, *Vcam1* and *Clu* are upregulated in both parts of the aorta, whereas *Fbln5* is significantly upregulated only in the thoracic and abdominal parts. Scale, log-transformed gene expression. Statistical significance was determined using a Wilcoxon rank-sum test. *P* value adjustment was performed using Bonferroni correction based on the total number of genes in the dataset. **c**, Representative immunofluorescence images of aortic sections 20 weeks after Western diet and Pcsk9 overexpression stained for PECAM1 (green) and VCAM1 (red). Scale bar, 100 μm. **d**, Quantification of the VCAM1/PECAM1 ratio (*n* = 3 independent mice per group). Values show mean ± s.d. Statistical significance was determined using Student’s *t*-test (two-tailed, unpaired)*.* Source data

Endothelial cells are known to participate in the inflammation in atherosclerotic lesions via adhesion receptors and paracrine signaling^35,36^. We found that the aortic arch of atherosclerotic *Tie2*^*iΔAEC*^ mice exhibits increased vascular cell adhesion molecule 1 (VCAM1) expression after 8 weeks of Western diet plus Pcsk9 overexpression (Fig. 4a). By comparing differentially expressed genes (Gene Expression Omnibus (GEO) database; Extended Data Fig. 5g) in the *Tie2*^*WT*^ and *Tie2*^*iΔAEC*^ mice, we found that the *Tie2*^*iΔAEC*^ mice express significantly more transcripts encoding *Vcam1* leukocyte adhesion receptor, *Clu* (clusterin) molecular chaperone and *Fbln5* (fibulin-5), which are involved in inflammatory processes and vascular remodeling^37–40^ (Fig. 4b and Supplementary Data 1). As expected, analysis using the differentially expressed genes for ligand–receptor protein pairs^41^ suggested that VCAM1 interacts with leukocyte integrins in our dataset (Supplementary Data 4). Immunostaining of aortic sections confirmed increased VCAM1 expression in the *Tie2*^*iΔAEC*^ versus *Tie2*^*WT*^ mice (Fig. 4c, d). We also found that transcripts encoding intercellular (leukocyte) adhesion molecule 1 (*Icam1)* were upregulated by the mere *Tie2* deletion in untreated mice (Extended Data Fig. 5e, f and Supplementary Data 5). These data indicate that *Tie2* deletion leads to vascular inflammation that promotes the recruitment of immune cells to the aorta during atherosclerosis.

### Silencing Tie2 in aortic fibroblasts promotes inflammation

**Fig. 5:**
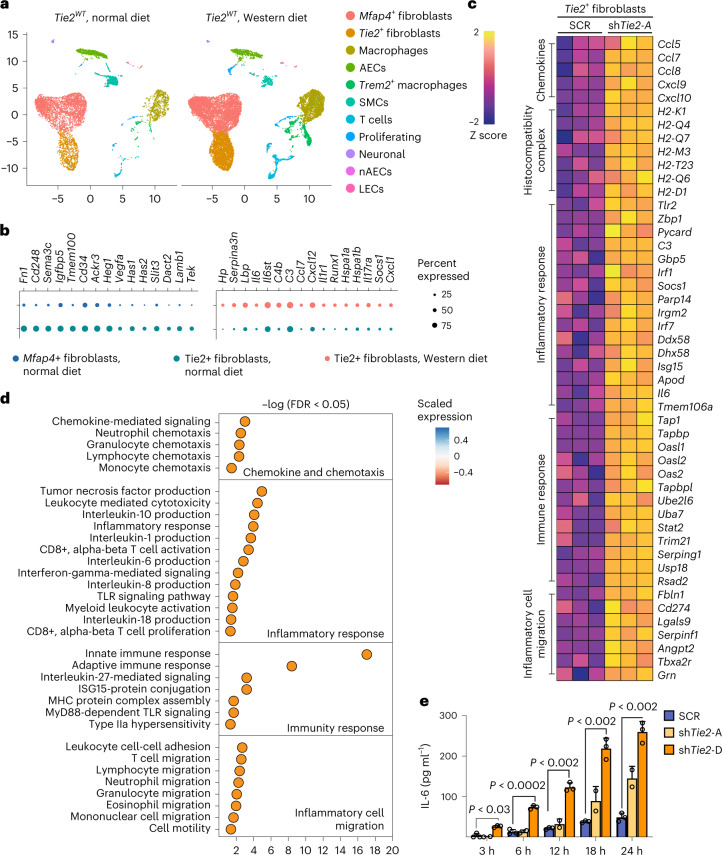
Tie2 restricts inflammatory signaling in the *Tie2*^+^ fibroblasts. **a**, UMAP plots of aortic cells from adult *Tie2*^*WT*^ mice that received normal diet and *Tie2*^*WT*^ mice after 20 weeks of Western diet. **b**, Dot plots showing top changed genes in the top regulated pathways in the Tie2^+^ fibroblasts. **c**, Heatmap showing upregulated genes related to inflammatory cell migration, immune response, inflammatory response, and chemokine and chemotaxis pathways upon Tie2 silencing. **d**, Gene ontology analysis on the GO terms that were upregulated in the Tie2-silenced Tie2^+^ fibroblasts. Bonferroni correction (multiple comparison test) was applied, followed by Benjamin–Hochberg adjusted *P* value or FDR values to plot the gene ontology. **e**, Quantification of IL-6 secreted in the Tie2^+^ fibroblasts after Tie2 silencing (*n* = 3 independent samples in SCR, *n* = 2 independent samples in shTie2-A and *n* = 3 independent samples in shTie2-D; 3 h, *P* = 0.0281, 6 h, *P* = 0.000102; 12 h, *P* = 3.21 × 10^−^^8^; 18 h, *P* = 2.16 × 10^−13^; 24 h, *P* = 3.5 × 10^−14^). Values show mean ± s.d. Statistical significance was determined using two-way ANOVA with Greenhouse–Geisser correction and Tukey’s multiple comparisons test. Source data

**Extended Data Fig. 7:**
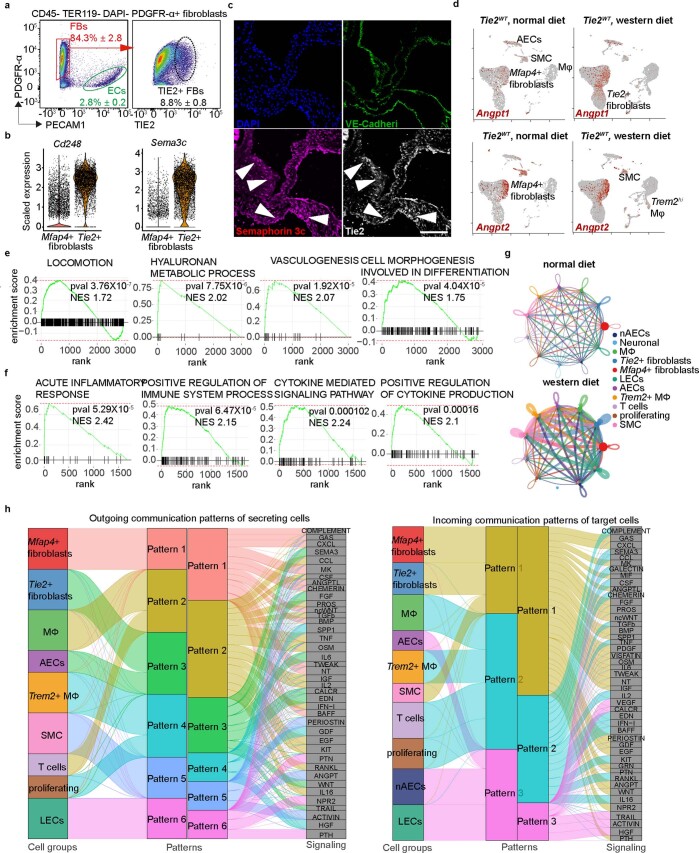
Identification of Tie2 expressing fibroblasts in the aortic wall. Representative flow cytometry analysis on the Tie2 expression of aortic cells using CD45, Ter119, PDGFRα and CD31 staining (*n* = 3 independent mice)**. b**. Violin plots showing *Cd248* and *Sema3c* expression in the Tie2+ fibroblasts and Mfap4+ fibroblasts as indicated in Fig.2a. **c**. Representative immunofluorescent images of Tie2+ fibroblasts in the aortic root and aortic arch. The sections were stained for PECAM1 (green), TIE2 (grey), SEMAPHORIN 3 C (red). Scale bar, 50 μm. **d**. Feature plots showing the expression of *Angpt1* and *Angpt2* in the aortic cells from the *Tie2*^*WT*^ mice and cells from aortic arch and thoracic plus abdominal aortas of the *Tie2*^*WT*^ mice after 20 weeks of Pcsk9 overexpression and Western diet. Note *Ang1* and Ang2 in *Mfap4* + fibroblasts and SMCs. **e-f**. Gene enrichment analysis showing pathways that are related the upregulated genes in the Tie2+ fibroblasts from mice fed with normal diet (**e**) and Western diet (**f**). *P* value adjustment is performed using a Benjamini-Hochberg (BH)-procedure as indicated in the fgsea package. **g**. Circle plot showing the number of interactions between any two cell groups in the aorta during atherosclerosis analyzed with CellChat. **h**. Alluvial plot visualizing the outgoing signaling patterns of secreting cells and the incoming signaling patterns of target cells, which shows the correspondence between the inferred patterns, cell groups and signaling pathways.

The Tie2^+^ fibroblasts discovered in the scRNA-seq analysis comprised 8.8% of aortic platelet-derived growth factor receptor-α-positive (PDGFRα^+^) fibroblasts. The Tie2^+^ fibroblasts were located in the subendothelial intimal layer and in the tunica adventitia, as indicated by immunofluorescence staining using antibodies against Tie2 and semaphorin 3C (Extended Data Fig. 7a–c). To understand the importance of Tie2 expression in the Tie2^+^ fibroblasts, we compared scRNA-seq profiles of aortic cells from *Tie2*^*WT*^ mice maintained under normal diet with the cells from *Tie2*^*WT*^ mice overexpressing Pcsk9 and fed a Western diet for 20 weeks (Fig. 5a). Bioinformatic analysis revealed that aortic *Angpt1* and *Angpt2* are expressed mainly by *Mfap4*^+^ fibroblasts and SMCs, but not by the AECs or Tie2^+^ fibroblasts (Extended Data Fig. 7d), suggesting that the *Mfap4*^+^ fibroblast-dervied or SMC-derived Angpt(s) could activate Tie2 receptors in the Tie2^+^ fibroblasts. Compared with *Mfap4*^+^ fibroblasts, the Tie2^+^ fibroblasts in mice fed a normal diet were enriched for transcripts related to locomotion, hyaluronan metabolic process, vasculogeneisis, and cell morphogenesis involved in differentiation, whereas transcripts of oxidative phosphorylation were downregulated (Fig. 5b, Extended Data Fig. 7e and Supplementary Data 6). Tie2^+^ fibroblasts isolated from the undeleted mice fed a Western diet showed upregulated pathways related to acute inflammatory response, positive regulation of immune system process, cytokine-mediated signaling pathway and positive regulation of cytokine production (Extended Data Fig. 7f and Supplementary Data 6). This suggests that Tie2^+^ fibroblasts exert an immunomodulatory role during atherosclerosis development.

In CellChat ligand–receptor analysis, Mfap4^+^ fibroblasts were identified as a dominant communication ‘hub’ in the aorta. The Tie2^+^ fibroblasts were predicted to produce or secrete 141 proteins and potentially form 132 ligand–receptor interactions during atherosclerosis (Extended Data Fig. 7g). Pattern recognition analysis revealed that Tie2^+^ fibroblasts and AECs together coordinate outgoing signals of the FGF, IL-6, WNT and SEMA3 pathways (pattern 3, Extended Data Fig. 7h). Tie2^+^ fibroblasts, Mfap4^+^ fibroblasts and SMCs were predicted to be capable of responding to incoming cytokines, such as CXCL, SPP1, PDGF, Angpt and FGF (pattern 1, Extended Data Fig. 7h).

**Extended Data Fig. 8:**
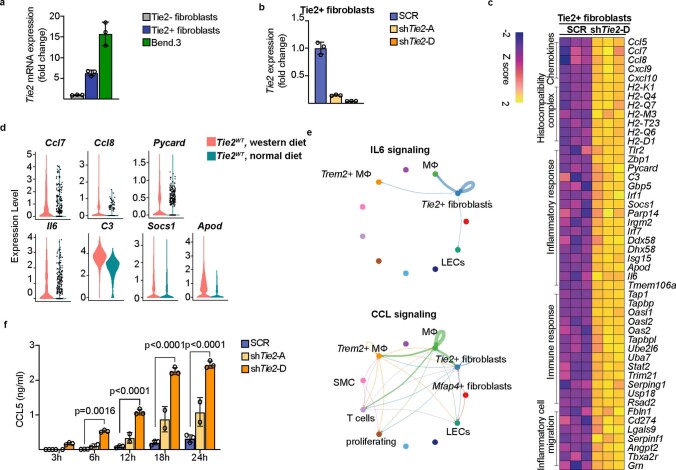
Effect of Tie2 silencing on gene expression in cultured Tie2 + FBs. **a**. Quantifications of *Tie2* (*Tek*) mRNA level in cultured Tie2+ FBs and Tie2- FBs (Passage 6) in comparison with murine endothelial cell line Bend.3 (*n* = 3 independent mice per group). **b**. Quantification of *Tie2* (*Tek*) mRNA level in the Tie2+ FBs transduced with shTie2-A or shTie2-D vs scramble (SCR) control (*n* = 3 independent mice per group). **c**. Heatmap showing upregulated transcripts related to inflammatory cell migration, immune response, inflammatory response, and chemokine and chemotaxis pathways in the Tie2-silenced Tie2+FBs. **d**. Violin plots showing the upregulation of *Ccl7*, *Ccl8*, *Pycard*, *C3*, *Socs1*, *Apod*, and *Il6* in the Tie2+ fibroblasts during feeding Western diet in *Tie2*^*WT*^ mice. **e**. Circle plot showing the significant communications in the IL6 signaling pathway and CCL signaling pathway analyzed using CellChat. **f**. Quantification of CCL5 secreted in the Tie2+ fibroblasts after Tie2 silencing (*n* = 3 independent samples in SCR, *n* = 2 independent samples in shTie2-A, *n* = 3 independent samples in shTie2-D; 6 h: *P* = 0.0016, 12 h: *P* = 3.59 × 10^−9^, 18 h: *P* = 3.0 × 10^−14^, 12 h: *P* = 2.9 × 10^−14^). Values show mean ± s.d. Statistical significance was determined using Two-way ANOVA with Geisser-Greenhouse correction and Turkey’s multiple comparisons test to the scramble control. Source data

To explore the intrinsic function of Tie2 in the Tie2^+^ fibroblasts, we used fluorscence-activated cell sorting (FACS) to isolate them from the aortas of several wild-type mice of the C57BL/6J strain and cultured them for six passages (Extended Data Fig. 8a). These cells were then transduced with two independent shTie2 lentiviral vectors, both of which reduced the *Tie2* mRNA by approximately 85–95%, or a scramble control vector (SCR) (Extended Data Fig. 8b). RNA-seq analysis showed that 16 genes were downregulated and 108 genes were upregulated in the shTie2-transfected versus SCR-transfected Tie2^+^ fibroblasts. Gene Ontology (GO) analysis indicated upregulation of transcripts related to inflammatory cell migration, inflammatory responses, immune responses, chemokines and chemotaxis (Fig. 5c, d and Extended Data Fig. 8c). This confirmed that Tie2 signaling regulates immunomodulatory responses in the Tie2^+^ fibroblasts. Furthermore, the inflammation-related transcripts encoding *Ccl7, Ccl8, Il-6, C3* (complement C3), *Socs1, Pycard* (Pyd and Card domain containing) and *ApoD* (apolipoprotein D), which were upregulated in the cultured Tie2-silenced Tie2^+^ fibroblasts, were also elevated in the Tie2^+^ fibroblasts from *Tie2*^*WT*^ mice fed Western diet for 20 weeks versus Tie2^+^ fibroblasts obtained from *Tie2*^*WT*^ mice maintained on regular chow diet (Extended Data Fig. 8c, d). These results are consistent with previous findings indicating that IL-6, CCL7 and CCL8 are inflammatory biomarkers in atherosclerosis, and that *C3* and *Socs1* are increased in atherosclerotic lesions^42–45^. The CellChat analysis furthermore suggested that Tie2^+^ fibroblasts coordinate outgoing IL-6 signaling, which targets macrophages and CCL signaling, thus affecting T cells and macrophages (Extended Data Fig. 8e). Enzyme-linked immunosorbent assay (ELISA) analysis confirmed that IL-6 and CCL5 secretion was increased in shTie2-silenced versus SCR-silenced Tie2^+^ fibroblasts (Fig. 5e and Extended Data Fig. 8f). These data suggest that the angiopoietin/Tie2 signals can alter immunomodulatory functions of the Tie2^+^ fibroblasts during atherosclerosis in mice.

### Endothelial Tie2 dominates Tie1 function in atherosclerosis

**Extended Data Fig. 9:**
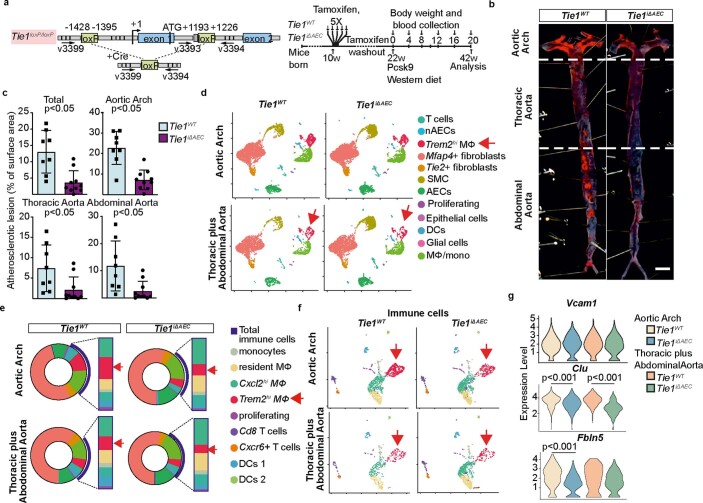
Tie1 deletion reduces atherosclerosis. **a**. Detailed schematic of the gene deletions. Numbers indicate positions of the loxP sites relative to the transcription start site, and names of the genotyping primers (right). Outline and time schedule (in weeks, w) of the experimental setup. **b**. Representative images of the *Tie1*-deleted aortas stained with Sudan IV (scale bar, 2 mm). **c**. Quantification of aortic lesion areas from the experiments (*n* = 8 independent mice per group, total: *P* = 0.0012, aortic arch: *P* = 0.0001, thoracic *P* = 0.024, abdominal *P* = 0.0098). Values show mean ± s.d. Statistical significance was determined using Student’s *t*-test (two tailed, unpaired). **d**. UMAP plots of aligned gene expression data in atheromatous aortic cells isolated from the aortic arch and thoracic plus abdominal aortas of the *Tie1*^*WT*^ (*n* = 4994 and *n* = 2696, respectively) and *Tie1*^*iΔAEC*^ (*n* = 6259 and *n* = 4892, respectively) mice after 20 weeks of w, partitioned into 12 distinct clusters. *Trem2*^*hi*^ macrophages (red arrow) showed the most obvious changes after *Tie1* deletion. **e**. Proportions of distinct clusters (**inner circle**) and total immune cells (**blue arch**) among the aortic cells. Frequencies of main immune cell types (macrophage subtypes, T cells, DCs, proliferating immune cells) (**column**). Frequencies of *Trem2*^*hi*^ macrophages are indicated with the red arrows. **f**. UMAP plot of immune clusters from the atheromatous aortic arch and thoracic plus abdominal aortas of the *Tie1*^*WT*^ and *Tie1*^*iΔAEC*^ mice, partitioned further into 9 sub-clusters. **g**. Violin plots showing *Vcam1*, *Clu and Fbln5* expression in the atheromatous aortic arch (*Clu*, p.adj=3.08×10^-8^), *Fbln5* p.adj=1.01×10^-6^) and thoracic plus abdominal aortas (*Clu*, p.adj = 1.28×10^-20^) of the *Tie1*^*WT*^ and *Tie1*^*iΔAEC*^ mice. Statistical significance was determined using a Wilcoxon rank-sum test. *P* value adjustment is performed using bonferroni correction based on the total number of genes in the dataset as indicated in the Seurat package. Source data

*Tie1* deletion is known to decrease atherosclerosis in ApoE^−/−^ mice by reducing vascular inflammation^16^. Our data indicated that *Tie2* deletion results in an opposite phenotype. Therefore, we analyzed the phenotypes of mice with a combined *Tie2* plus *Tie1* gene deletion in atherosclerosis-promoting conditions. First, we generated *BmxCre*^*ERT2*^;*Tie1*^*fl/fl*^ mice to obtain *Tie1*^*iΔAEC*^ mice by tamoxifen administration. Consistently with the results obtained by Woo et al.^16^, we found fewer atherosclerotic plaques and leukocytes (including macrophage and monocytes, *Trem2*^*hi*^ macrophages, T cells, and dendritic cells) in the aortic arch of *Tie1*-deleted mice than littermate control mice (22.2% versus 30.3%; Extended Data Fig. 9a–e). A similar, but less prominent decrease occurred in the thoracic plus abdominal part of the aorta (21.7% versus 23.3%; Extended Data Fig. 9d, e). There were much fewer *Trem2*^*hi*^ macrophages in the *Tie1*-deleted than in littermate control aortas (4.4% versus 10.3% in the aortic arch, 3.6% versus 5.7% in the thoracic and abdominal aorta; Extended Data Fig. 9e, f). Importantly, unlike *Tie2* deletion, *Tie1* deletion did not affect *Vcam1* expression, and it decreased the expression of *Clu and Fbln5* (Extended Data Fig. 9g and Supplementary Data 7; compare with Fig. 4b), suggesting that Tie1 and Tie2 have opposite effects on inflammatory responses involved in atherosclerosis.

**Fig. 6:**
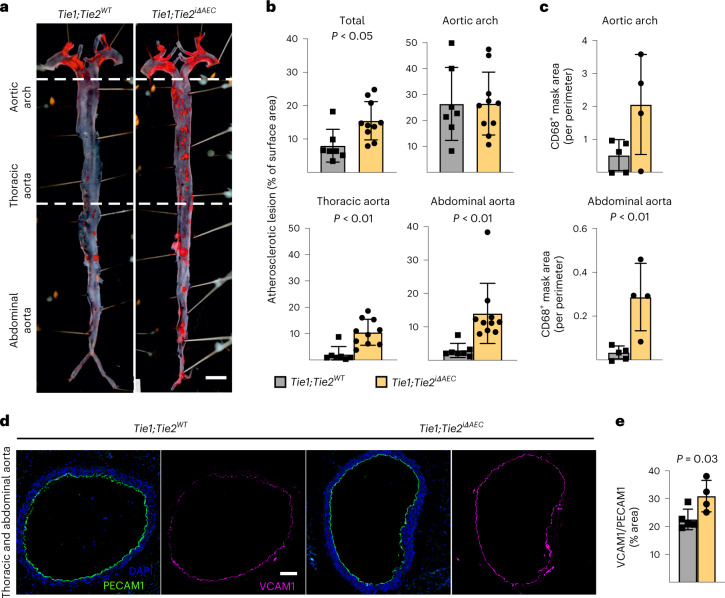
Tie2 function dominates over Tie1 function in atherosclerosis development. **a**, Representative images of *Tie1*^*WT*^*Tie2*^*WT*^ and *Tie1;Tie2*^*iΔAEC*^ (*n* = 7 independent *Tie1*^*WT*^*Tie2*^*WT*^ mice and *n* = 10 independent *Tie1;Tie2*^*iΔAEC*^ mice) aortas stained with Sudan IV (scale bar, 2 mm). **b**, Quantification of lesion areas in the *Tie1*^*WT*^*Tie2*^*WT*^ and *Tie1;Tie2*^*iΔAEC*^ aortas (*n* = 7 independent *Tie1*^*WT*^*Tie2*^*WT*^ mice and *n* = 10 independent *Tie1;Tie2*^*iΔAEC*^ mice over two independent experiments; total, *P* = 0.0132; thoracic aorta, *P* = 0.0012; abdominal aorta, *P* = 0.0063). Values show mean ± s.d. Statistical significance was determined using Student’s *t*-test (two-tailed, unpaired). **c**, Quantification of CD68^+^ area (*n* = 5 independent *Tie1*^*WT*^*Tie2*^*WT*^ mice and *n* = 4 independent *Tie1;Tie2*^*iΔAEC*^ mice; *P* = 0.0081). Values show mean ± s.d. Statistical significance was determined using Student’s *t*-test (two-tailed, unpaired). **d**, Representative immunofluorescence images of the *Tie1*^*WT*^*Tie2*^*WT*^ and *Tie1;Tie2*^*iΔAEC*^ aorta sections stained for PECAM1 (green), DAPI (blue) and VCAM1 (red). **e**, Quantifications of VCAM1 in the *Tie1*^*WT*^*Tie2*^*WT*^ and *Tie1;Tie2*^*iΔAEC*^ aortas (*n* = 5 independent *Tie1*^*WT*^*Tie2*^*WT*^ mice and *n* = 4 independent *Tie1;Tie2*^*iΔAEC*^ mice). Values show mean ± s.d. Statistical significance was determined using Student’s *t*-test (two-tailed, unpaired). Scale bar, 100 μm. Source data

**Extended Data Fig. 10:**
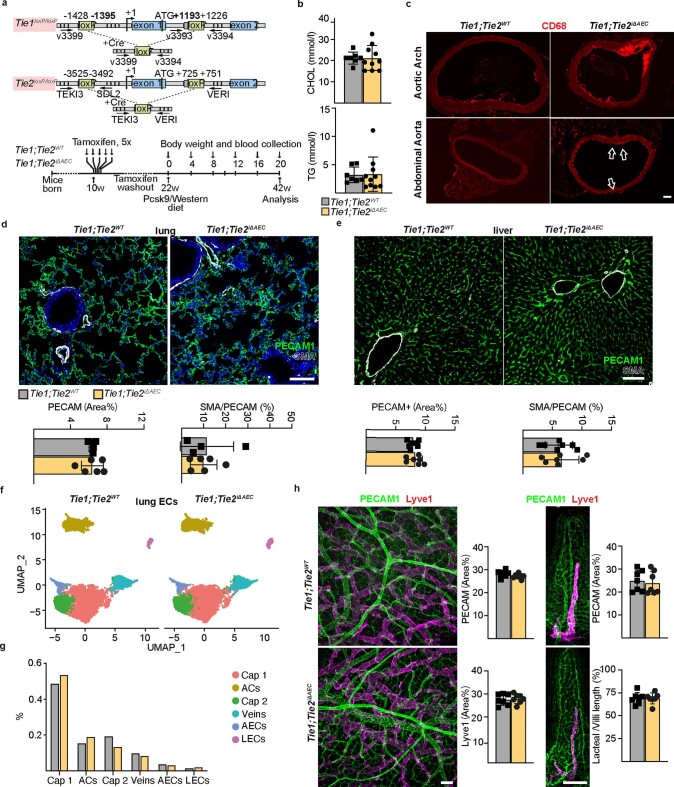
*Tie1;Tie2* deletions in the aorta. **a**. Detailed schematic of the gene deletions. Numbers indicate positions of the loxP sites relative to the transcription start site, and names of the genotyping primers. **b**. Cholesterol (CHOL) and triglyceride (TG) concentrations in serum samples from the *Tie1*^*WT*^*Tie2*^*WT*^ and *Tie1;Tie2*^*iΔAEC*^ mice 20 weeks after Pcsk9 and Western diet (*n* = 7 independent *Tie1*^*WT*^*Tie2*^*WT*^ mice and *n* = 10 independent *Tie1;Tie2*^*iΔAEC*^ mice). **c**. Representative immunofluorescent images of aorta sections stained for CD68 (red). **d**. Representative immunofluorescent images of the lung sections from the *Tie1*^*WT*^*Tie2*^*WT*^ and *Tie1;Tie2*^*iΔAEC*^ mice stained for PECAM1 (green), DAPI (blue), and SMA (white). Quantification of PECAM1 and SMA/PECAM1 ratio (*n* = 4 independent *Tie1*^*WT*^*Tie2*^*WT*^ mice and *n* = 6 independent *Tie1;Tie*^*iΔAEC*^ mice). **e**. Representative immunofluorescent images of liver sections stained for PECAM1 (green), DAPI (blue), and SMA (white). Quantification of PECAM1 and SMA/PECAM1 ratio (*n* = 7 independent mice per group). **f**. UMAP plot of lung endothelial clusters from the *Tie1*^*WT*^*Tie2*^*WT*^ and *Tie1;Tie2*^*iΔAEC*^ mice 20 weeks after Western diet and Pcsk9 overexpression, partitioned further into 6 clusters. **g**. Proportions of distinct clusters among the lung endothelial cells. **h**. Representative immunofluorescence images of the intestine stained for PECAM1 (green) and Lyve1 (red). Quantifications of PECAM1, Lyve1 and lacteal/villus length ratio (*n* = 7 independent mice per group). Scale bar, 100 μm. Values show mean ± s.d. Source data

To understand how arterial Tie1 and Tie2 together affect atherosclerosis development, we next generated *BmxCre*^*ERT2*^;*Tie1*^*fl/fl*^;*Tie2*^*fl/fl*^ mice (*Tie1;Tie2*^*iΔAEC*^). Interestingly, similarly as in the *Tie2*-deleted mice, the *Tie1;Tie2*^*iΔAEC*^ mice exhibited more atherosclerotic plaques than *Tie1;Tie2*^*WT*^ mice in the thoracic and abdominal parts of the aorta after 20 weeks of treatment with AAV-Pcsk9 plus Western diet (Fig. 6a, b and Extended Data Figs. 2d and 10a). This indicated that arterial Tie2 function dominates over Tie1 function in atherosclerosis development. We did not observe any difference in the total serum cholesterol or triglyceride levels between the *Tie1;Tie2*^*WT*^ and *Tie1;Tie2*^*iΔAEC*^ mice (Extended Data Fig. 10b). As in the *Tie2*^*iΔAEC*^ aortas, CD68^+^ macrophages were increased in the *Tie1;Tie2*^*iΔAEC*^ aortas, indicating increased inflammatory cell infiltration after arterial *Tie1* and *Tie2* deletion, especially in the thoracic plus abdominal parts of the aorta (Fig. 6c and Extended Data Fig. 10c). Immunostaining showed increased expression of leukocyte adhesion molecule VCAM1 in the aortic endothelium of the *Tie1;Tie2*^*iΔAEC*^ versus *Tie1;Tie2*^*WT*^ mice (Fig. 6d, e).

As in the *Tie2*^*iΔAEC*^ mice, we found that PECAM1^+^ or smooth muscle actin (SMA)/PECAM1^+^ vascular areas were similar in the lungs or livers of the *Tie1;Tie2*^*iΔAEC*^ and *Tie1;Tie2*^*WT*^ mice after 20 weeks of treatment with AAV-Pcsk9 plus Western diet (Extended Data Fig. 10d, e). Furthermore, our extended scRNA-seq analysis indicated that pulmonary endothelial subclusters were not affected in these mice (Extended Data Fig. 10f, g). Because lymphatic vessels are involved in fat absorption from the gut, we also confirmed that the total PECAM1^+^ blood vascular and lymphatic vessel endothelial hyaluronan receptor 1^+^ (LYVE1^+^) lymphatic vessel area in the intestine were unaltered (Extended Data Fig. 10h).

**Fig. 7:**
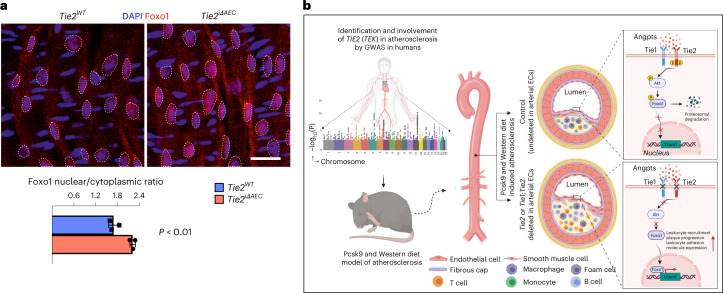
*Tie2* deletion promotes nuclear localization of Foxo1. **a**, Representative en face images of *Tie2*^*WT*^ and *Tie2*^*iΔAEC*^ (*n* = 3 per group) aortas stained for Foxo1 (red) and DAPI (blue) (top). Scale bar, 20 μm. Quantification of nuclear versus cytoplasmic Foxo1 expression in the endothelial cells (dashed lines) (*n* = 3 independent mice per group; *P* = 0.0044) (bottom). Values show mean ± s.d. Statistical significance was determined using Student’s *t*-test (two-tailed, unpaired). **b**, Schematic showing a summary of the main results. GWAS analysis indicates that Tie2 activity is involved in human CAD via disruption of Tie–Akt signaling axis and via inhibition of Foxo1 nuclear localization and transcriptional activity. When Tie2 alone or the whole Tie1;Tie2 receptor complex is deleted from the aortic endothelium, VCAM1 expression is induced via Foxo1 transcriptional transactivation and blood leukocytes adhere to the aortic endothelium, which promotes atheroma formation. ECs, endothelial cells. Source data

Next, we sought to identify the link between Tie2 activity and VCAM1 expression in the AECs. We considered that Tie1 can affect angiopoietin-induced Tie2 phosphorylation^46^. On the other hand, Tie2 activation can inhibit nuclear localization of forkhead box O1 (Foxo1) transcription factor via PI3K/Akt-dependent phosphorylation, relocating Foxo1 to the cytoplasm^11,12,47^. Therefore, we analyzed the ratio between nuclear versus cytoplasmic localization of Foxo1 in en face-stained thoracic and abdominal aortas after tamoxifen-induced *Tie2* deletion for 1 week. The immunofluorescence staining indicated that *Tie2* deletion results in increased nuclear localization of Foxo1 in the AECs of the thoracic aorta (Fig. 7a). As Foxo1 can directly bind to and activate the VCAM1 upstream promoter region that contains two Foxo1-responsive DNA elements^48,49^, these findings indicated a linkage between *Tie2* deletion, Foxo1 nuclear localization and increased VCAM1 expression. This is in agreement with our data that Vcam1 expression was significantly upregulated in the thoracic and abdominal parts of aortas from the *Tie2*^*iΔAEC*^ mice where we detected the increase in atherosclerotic plaques.

Collectively, our data indicate that an organ-specific vascular inflammatory response in the aorta is an important contributor to the increased atherosclerosis after arterial *Tie2* or *Tie1* plus *Tie2* deletion (Fig. 7b), and that of these two receptors, Tie2 is the dominant receptor that counteracts inflammation and atherosclerosis in part via modulation of Foxo1 subcellular localization.

## Discussion

In this study, we provide evidence that variation in *TIE2* expression is associated with human coronary atherosclerosis by taking advantage of the increased power of discovery provided by meta-analysis of GWAS^23–27^. We found a strong association of a common intronic *TIE2* variant (rs1322052) with CAD. This was supported by our finding that arterial *Tie2* protects from atherosclerosis in a Western diet mouse atherosclerosis model in which the LDL receptor is downregulated by AAV-mediated expression of an activated form of Pcsk9. In this model, Tie2 function dominates in atherosclerosis over Tie1 function.

Interestingly, our data also revealed the existence of Tie2-expressing fibroblasts in the aortic wall. The Tie2^+^ fibroblasts are enriched for immunomodulatory transcripts and, like AECs, they have a capability to respond to Angpt(s) in the vessel wall. CCL5 promotes the recruitment of monocytes during early stages of atherosclerosis. Neutralization of IL-6 and CCL5 has been suggested to be beneficial in cardiovascular disease, including atherosclerosis^45,50^. In mice, selective disruption of CCL5–CXCL4 complexes with cyclic peptides decreased atherosclerosis development^51^. However, the IL-6 and CCL5-producing and responsive cells have not yet been fully characterized; evidence so far suggests that SMCs secrete both IL-6 and CCL5, and activated platelets release CCL5 in atherosclerosis^45,50^. IL-6 is one of the main outgoing signals in the Tie2^+^ fibroblasts, in which IL-6 and CCL5 secretion is increased by Tie2 targeting in vitro, suggesting that Tie2 is atheroprotective also in the Tie2^+^ fibroblasts. However, published scRNA-seq datasets reveal relatively low frequency of Tie2^+^ fibroblasts in human coronary artery^52^ or carotid artery^53^, suggesting species-specific Tie2 signaling in fibroblasts.

Our results demonstrate increased margination of myeloid cells along the atherosclerotic carotid artery wall at vessel branchpoints in *Tie2*^*iΔAEC*^ versus *Tie2*^*WT*^ mice. The increased microbead labeling of the same areas probably reflects the difference in the number of phagocytes in the emerging lesions. These findings suggest that the increased endothelial VCAM1 adhesion receptor in the *Tie2*-deleted AECs promotes leukocyte accumulation into the lesions by interacting with leukocyte integrins. Elevated VCAM1 expression is accompanied by increased macrophage and T cell numbers also in human atherosclerotic plaques, and VCAM1 blocking antibodies have been reported to abrogate leukocyte adhesion to atherosclerotic endothelium^54–56^. In contrast, deficiency of ICAM1 either alone or in combination with VCAM1 deficiency did not alter the formation of atherosclerotic lesions in LDL receptor-deficient mice^37^.

Our finding that deletion of *Tie2* increases and *Tie1* deletion decreases vascular inflammation is consistent with the previous finding by Woo et al.^16^ that Tie1 attenuation downregulates pro-inflammatory genes and IκB expression. Previous studies have shown that Tie1 can regulate Tie2 phosphorylation and activation of downstream signals, but Tie1 activation by angiopoietins is Tie2 dependent^11,16,46^. Our data support the concept that Tie2 signaling in the arteries suppresses VCAM1 expression at least in part by inhibiting the localization of Foxo1 in AEC nuclei, where it can directly activate VCAM1 expression^48,49^. These data are consistent with the results presented by Tsuchiya et al.^49^, showing that genetic deletion of all three *Foxo* genes led to marked decrease of insulin-dependent Akt phosphorylation, lower levels of VCAM1 in endothelial cells and prevented atherosclerosis in mice^49^. Regarding the reciprocal activity of Tie1 and Tie2 in atherosclerosis, it should be noted that deletion of either one from the AECs did not significantly affect the expression of the other *Tie* gene (Extended Data Fig. 2d). Thus, Tie1 promotes and Tie2 protects from atherosclerosis.

The results of our scRNA-seq analysis of immune cells in the atheromatous aortas resemble those in published datasets from mice and humans^29,31,57^. The inflammatory Cxcl2^hi^ macrophages in the *Tie2*^*iΔAEC*^ versus *Tie2*^*WT*^ mice exhibited a gene expression profile related to increased leukocyte cell adhesion, which also supports our finding of increased arterial VCAM1 expression and myeloid cell adhesion to the arterial wall. The increase in the frequency of *Ccl5*^+^ T cells among the total T cells in the *Tie2*-deleted atheromatous aortas is interesting, as human T cells were suggested to activate macrophages and to promote plaque vulnerability via CCL5–CCR5 and Toll-like receptor/versican signaling^33,57^^,^. Together, the AECs and fibroblasts may create a vascular inflammatory phenotype that recruits immune cells, including T cells and myeloid cells, via enhanced expression of leukocyte adhesion and inflammatory cytokines. The resulting increase in inflammatory cell homing to the aortic wall would thus be the major driving force behind the increased atherosclerosis in the *Tie2*^*iΔAEC*^ mice.

In conclusion, our results support the concept that the two endothelial Tie receptors function in an opposite manner during atherosclerosis and that, of the two, Tie2 is the dominant receptor in the development of atherosclerosis. As both Tie receptors can be modulated via their extracellular domains that are available in vessel lumen, they provide vascular targets for counteracting inflammation via Foxo1 transcriptional activity, and hopefully eventually for treatment of atherosclerosis.

## Methods

### Mice and tissues

All mouse experiments were approved by the Committee for Animal Experiments of the District of Southern Finland. The *Tie1*^*fl/fl*^ (ref. ^58^), *Tie2*^*fl/fl*^ (ref. ^59^), *BmxCre*^*ERT2*^ (ref. ^60^) and *Bmx*^*LacZ*^ (ref. ^61^) mouse strains have been described previously. All experiments were conducted in the C57BL/6J genetic background. Supplementary Table 1 shows the list of primers used for genotyping. Mice were provided with water and food ad libitum and on a 12 h/12 h light/dark cycle and kept at 22 ± 2 °C with relative humidity of 55 ± 10%. For induction of Cre-mediated recombination in adult mice, five consecutive intragastric doses of tamoxifen (Sigma-Aldrich; 20 mg ml^−1^ dissolved in corn oil, 100 µl per mouse) were administered to male mice when the mice reached the age of 10 weeks. Age and gender matched mice were used as controls. Adult *Tie1*/*2* single-deleted or double-deleted mice with their controls (at 22 weeks of age) received a single dose of a recombinant AAV serotype 9 (AAV9) expressing Pcsk9 (2 × 10^11^ viral particles per mouse) to promote atherosclerosis via LDL receptor downregulation^62^. Simultaneously with AAV9-Pcsk9 treatment, the mice were put on Western diet (Research Diets, D12079BI) for a period up to 20 weeks.

### GWAS and eQTL colocalization analysis

Summary statistics were downloaded for the TIE2 (TEK) locus from the CVD hugeamd portal (broadcvdi.org, 2021 11/30), converted to hg38 using the USCS liftOver tool. GWAS data were imported to the ezQTL tool^63^, which performed colocalization analysis with GTEx v8 Tibial Artery data using HyPrColoc^64^ ±75 kilobases (kb) around the variant to inquire (rs1322052).

### CRISPR experiments

To identify *cis*-regulatory elements at the rs1322052 variant site, we interrogated open chromatin (assay for transposase-accessible chromatin using sequencing, ATAC-seq) and histone mark data (H3K27ac) in published data from teloHAECs^65^. Using this information, guide RNAs (gRNAs) were designed for deletion of the variant-containing enhancer element, while making sure that no exonic regions were deleted. The CRISPR inhibition (CRISPRi) experiments were used to target the exact vicinity of the rs1322052 variant site.

TeloHAECs (American Type Culture Collection (ATCC), CRL-4052) were used for the CRISPR deletion and CRISPRi experiments. The cells were maintained in Vascular Cell Basal Medium (ATCC, PCS-100-030), supplemented with Vascular Endothelial Cell Growth Kit-VEGF (ATCC, PCS-100-041) and 100 IU ml^−1^ penicillin/100 μg ml^−1^ streptomycin. The CRISPR deletion experiments were performed using the standard protocol of Integrated DNA Technologies (IDT) and the Neon Transfection System (Thermo Fisher Scientific, MPK5000). In brief, the Alt-R CRISPR-Cas9 crRNA/gRNA (Negative Control crRNA #1, 1072544; L1, GCAGGTTTAGTGAGCTCCAAGTTTTAGAGCTATGCT; R1, GGAGTTGTAGCTTCCATACGGTTTTAGAGCTATGCT; R2, AGTTGTACCTACAAGAAAGTGTTTTAGAGCTATGCT; Extended Data Fig. 1c), Alt-R CRISPR-Cas9, tracrRNA (IDT, 1072533), Alt-R CRISPR-Cas9 Electroporation Enhancer (IDT, 1075916) and Alt-R S.p. HiFi Cas9 Nuclease V3 (100 µg; IDT, 1081060) were diluted fresh before each experiment. The ribonucleoprotein (RNP) complex was prepared for each treatment by following the manufacturer’s guidelines. Fifty thousand teloHAECs, specific RNP complex, and Alt-R Cas9 electroporation enhancer were mixed before loading into 10 µl of the Neon Transfection System kit. Electroporation was performed using the following settings: voltage, 1,350 V; width, 30 ms; pulses, 1 pulse. The newly transfected cells were inoculated immediately to pre-warmed complete cell culture medium, and three consecutive transfection reactions were performed for each well of a 12-well plate. Finally, cells were allowed to grow for 48 h before collection. The genomic DNA and RNA were collected from the same sample using a Quick-DNA/RNA Miniprep Plus Kit (Zymo Research, D7003). The CRISPR–RNP deletion of the specific target regions was confirmed by PCR followed by agarose gel electrophoresis using genomic DNA and target-specific primer pairs (forward, CCTTTCCCTGTTGTGTGTTC; reverse, CCGGCACAAGAATTACACTACC).

To perform CRISPRi, we generated teloHAECs stably expressing dCAS9-KRAB using a lentivirus-based approach. The lentiviral vector dCas9-KRAB-MeCP2-2A-Blast was a gift from A. Califano (Addgene, 122205). The viral vector was transduced to teloHAECs at multiplicity of infection (MOI) 20, screened by Blasticidin S HCL (Corning, Fisher Scientific) selection (6 µg ml^−1^) and stored in liquid nitrogen for future use. The gRNA (GTATGGACTTAGCAACAGAA) targeting the site 2 base pairs (bp) upstream of rs1322052 was duplexed with tracrRNA (named ‘crRNA:tracrRNA duplex’), and the transfection reaction was performed as described above with the exception of absence of Alt-R S.p. HiFi Cas9 Nuclease V3 in the mixture. The RNA was extracted using a Monarch Total RNA Miniprep Kit (New England Biolabs) 48 h after transfection.

To measure the effect of CRISPR deletion and CRISPRi on *TIE2* gene expression, RNA was reverse-transcribed into cDNA using a RevertAid First Strand cDNA Synthesis Kit (Themo Scientific), and qPCR was performed using KiCqStart SYBR Green qPCR ReadyMix (Sigma-Aldrich). Relative *TIE2* gene expression was calculated in comparison with *RPLP0* housekeeping gene expression using the ΔΔCt method. The following qPCR primers were used (Sigma-Aldrich): FH1_TEK, AAGACCTACGTGAATACCAC and BH1_TEK, GAAACAGAGGGTATACAGATG; FH1_RPLP0, AATCTCCAGGGGCACCATT and BH1_RPLP0, CGCTGGCTCCCACTTTGT.

### Cloning and AAV production

The D377Y mutation was introduced into the *Pcsk9* open reading frame, which was then cloned into the AAV plasmid vector psubCMV-WPRE. The AAVs were produced by plasmid transfection of 293T cells and purified by step gradient ultracentrifugation, as described previously^66^.

### Blood sampling and analysis of serum triglyceride and cholesterol

Blood samples were taken every 2–4 weeks by bleeding the mouse saphenous vein and collecting approximately 70 μl of blood from every mouse. Total triglyceride and cholesterol levels in serum samples were analyzed by using Thermo Fisher Scientific kits (981301 and 981812, respectively).

### Preparation of single-cell suspension and cell sorting

Aortas were collected in PBS, and their surrounding adipose tissue was removed. The aortas were then minced into pieces and digested with a mixture of collagenase types I, II and IV (Gibco, 17100-017, 17101-015 and 17104-019, respectively), 1 mg ml^−1^ each, in Dulbecco’s phosphate-buffered saline (DPBS) for 30 min at 37 °C. Single-cell suspensions from lungs were prepared by enzymatic digestion with a mixture of collagenase H, dispase II and DNase I in PBS with 1% BSA for 15 min at 37 °C. After digestion, the cell suspensions were filtered through a 70 μm cell strainer. Red blood cells were lysed in ACK Lysing Buffer (Gibco, A10492-01). The isolated aortic cells were incubated with Fc block for 5 min on ice (BD Biosciences, 553141), followed by labeling with the fluorescent antibody conjugates Tie2–PE, CD31–FITC, PDGFRα–PE-Cy7, CD45–Pacific Blue and Ter119–Pacific Blue (all diluted 1:100) in DPBS supplemented with 2% heat-inactivated fetal calf serum (FCS) and 1 mM EDTA (FACS buffer) for 30 min on ice. The lung cells were similarly labeled with Ter119–FITC and CD31–APC.

Bone marrow cells were isolated by flushing the long bones using Ca^2+^-free and Mg^2+^-free HBSS (HBSS-free) with 2% heat-inactivated FCS. Spleen cells were obtained by crushing the spleen between two glass slides. The cells were dissociated into a single-cell suspension by gently passing them through a 25-gauge needle and then filtering them through a 70 μm nylon mesh. Red blood cells from bone marrow, spleen, aorta and peripheral blood were depleted using ACK lysing buffer. Cells were analyzed using anti-CD34, anti-CD135, anti-CD127, anti-Fc receptor, anti-Sca1, anti-c-kit, anti-Ter119, anti-B220, anti-Gr1, anti-CD11b, anti-CD3e and anti-F4/80 (all diluted 1:100) in Ca^2+^-free and Mg^2+^-free HBSS (HBSS-free) with 2% heat-inactivated FCS. 4,6-diamidino-2-phenylindole (DAPI) staining was used to exclude dead cells. Supplementary Table 2 shows the list of antibodies used. After the incubation, the cells were washed once with FACS buffer and filtered through a FACS tube filter (30 µm mesh size). Analysis and sorting were performed using a FACSAria II flow cytometer. All data were analyzed using FlowJo v10 software (Tree Star).

### scRNA-seq

Isolated aortic cells, bone marrow cells or lung endothelial cells from 2–3 individual *Tie2*^*WT*^*, Tie2*^*iΔAEC*^, *Tie1*^*WT*^*Tie2*^*WT*^ and *Tie1;Tie2*^*iΔAEC*^ mice were pooled together, resuspended in 0.04% BSA in DPBS as one sample per genotype and then analyzed using a Chromium Single Cell 3′ RNA sequencing system (Reagent Kit v3.1, 10x Genomics). Cells were loaded into Chromium Single Cell Chip aiming at capture of 10,000 cells per sample. Sample libraries were sequenced using an Illumina NovaSeq 6000 system S1 flow cell with the following read lengths: read 1, 28 bp; i7 index, 8 bp; i5 index, 0 bp; and read 2, 89 bp. Isolated aortic cells from 2–3 individual *Tie1*^*WT*^ and *Tie1*^*iΔAEC*^ mice were pooled together and analyzed using the Chromium Single Cell 3′ RNA sequencing system (Reagent Kit v2.1). Read lengths for the aortic cells from *Tie1*^*WT*^ and *Tie1*^*iΔAEC*^ mice were as follows: read 1, 26 bp; i7 index, 8 bp; i5 index, 0 bp; and read 2, 91 bp.

Raw sequencing data were processed using the Cell Ranger pipeline (10x Genomics). To reduce any potential effects due to ambient RNA contamination, we applied the SoupX^67^ method. To estimate ambient RNA fraction for each lane of 10x Genomics Chromium data, we ran SoupX using the Cell Ranger clustering information and the following lists of genes: *Hba*-*a1* and *Hba-a2* (hemoglobin genes); *Lyz2* and *Trem2* (macrophage genes); *Acta2* and *Tagln* (SMC genes); *Cdh5* and *Pecam1* (endothelial cell genes); *Cd3d, Cd8b1* and *Nkg7* (natural killer and T cell genes); *Cd79a, Igkc* and Ighm (B cell genes); and *Dcn, Sperinf1, Fbn1, ApoE* and *Igfbp5* (fibroblast genes). After estimating the contamination, the expression profiles were corrected using the SoupX function adjust Counts with the ‘round to integer’ option enabled. Count tables were loaded into R and processed with DoubletFinder^68^ and further using the Seurat toolkit v3.2.0^69,70^, according to the instructions on the Satija lab website (https://satijalab.org/seurat). Cells with fewer than 300 distinct genes observed or more than 7% of mitochondrial genes were excluded. Gene expression was normalized using logNormalization in Seurat. Cell cycle effects were not regressed. Features for integration among the datasets were obtained using the IntegrationData() function, and anchors were identified using FindIntegrationAnchors(). Based on the principal component elbow plot, 30 principal components were chosen for subspace alignment for the integration with IntegrationData(). We used a graph-based clustering approach using k-nearest neighbors with Jaccard similarity and Louvain in Seurat and visualized the data using uniform manifold approximation and projection (UMAP) with resolution 0.1. UMAP plots and a list of differentially expressed genes in the clusters were generated using the Bonferroni-corrected Wilcoxon rank-sum test implemented in the FindAllMarkers() function, as instructed by the Seurat vignettes. Immune cell clusters were subset and re-clustered using resolution 0.2. Differentially expressed genes were computed from the same aortic region in each cluster using the Bonferroni-corrected Wilcoxon rank-sum test implemented in the FindMarkers function between samples; genes with adjusted *P* values of greater than 0.05 were excluded.

To compare our Seurat-derived cell-clustering identity of immune cells, we used SingleR (v1.8.1)^34^ according to the instructions. Average expression in the published annotated cells were used as references. The correlated annotations for each single cell were then added to the Seurat object metadata. We used gene set enrichment analysis (GSEA; fGSEA v1.20.0) with MSigDB gene sets^71–73^ to identify pathways with upregulated or downregulated gene expression in cell clusters from the samples. For GSEA, we used the pre-ranked analysis mode, with transcripts ranked according to Wilcoxon rank-sum test (log_2_(fold change)) for comparison. Before running the fgsea test, we placed the most significantly upregulated genes at the top of the ranked list, and the most downregulated genes at the bottom.

The Seurat object was loaded into R, and the CellChat object was created using the normalized count data and cell group information from the Seurat object. The CellChat object was further analyzed using CellChat toolkit (v1.4.0)^74^ for ligand–receptor and pathway analysis according to the instructions. For the cell–cell communication analysis, the CellChatDB.mouse ‘Secreated Signaling’ database was used. The overexpressed ligands or receptors in one cell group were identified using the identifyOverexpressedGenes() and identifyOverexpressedInteractions() functions. The communication probability was computed using the computeCommunProb() function. Cell groups containing less than ten cells were filtered out from the analysis using the filterCommunication() function. We then calculated the communication probability on the signaling pathway with CellChat using the computeCommunProbPathway() function. The cell–cell communication network was then aggregated using the aggregateNet() function. The chosen signaling pathway was then visualized using a circle plot or chord diagram. The communiation patterns on Tie2^+^ fibroblasts and the signaling pathways that coordinate together were identified with the selectK() function and visualized using a river plot.

### Intravital microscopy

At week 4 after initiation of the atherogenic diet, circulating monocytes were labeled by intravenous injection (100 µl per mouse) of 1 µm Fluoresbrite green fluorescent (YG) plain microspheres (Polysciences), 3 days after clodronate liposome (Liposoma) injection. Eight weeks after initiation of high-fat diet feeding, mice were anesthesized, and an antibody that binds CD11b (clone M1/70, 1 µg, PE) was administered intravenously. The carotid artery was exposed, and the interaction of myeloid cells with the carotid artery was recorded using a ×10 water-dipping lens.

### Isolation, culture and gene silencing of aortic Tie2^+^ fibroblasts

Tie2^+^ fibroblasts (Tie2^+^Pdgfr-α^+^CD31^−^CD45^−^TER119^−^DAPI^−^) from the aortas were FACS sorted, seeded on the 0.1% gelatin-coated culture dishes (containing RPMI 1640 supplemented with 10% FCS, 1% L-glutamine, 1% penicillin/streptomycin, 50 µM β-ME, 1% non-essential amino acids and 2 ng ml^−1^ recombinant human FGF) and cultured until passage six.

For the RNA silencing approach, cultured Tie2^+^ fibroblasts were transfected with the lentiviral vectors encoding mouse Tie2 (shTie2-A or shTie2-D; clone IDs, TRCN0000023554 and TRCN0000023557; https://portals.broadinstitute.org/gpp/public/clone/search). After 24 h, 2 µg ml^−1^ puromycin was added for 48 h, and subsequently at 10 µg ml^−1^ for 12 h, followed by incubation in antibiotic-free growth medium for 48 h, until the analysis.

### RNA-seq of aortic Tie2^+^ fibroblasts

Total RNA was purified using a NucleoSpin RNA Mini kit (Macherey-Nagel, 740955.50), and RNA quality was determined using an Agilent 2100 bioanalyzer. A BGI DNBseq Stranded mRNA kit was then used to generate libraries, and 20 million paired-end 100 (PE 100) reads were sequenced in the MGISEQ-2000 platform (BGI). The paired-end reads were aligned to the GRCm38.95 genome using HISAT2 (v2.2.1)^75^ and counted using the HTSeq tool package (v0.13.5)^76^. Differential gene expression analysis was performed using the DESeq2 Bioconductor package (v3.16)^77^, and the Benjamini–Hochberg multiple correction test was applied to control false discovery rate (FDR). Differentially expressed genes with an adjusted *P* value cutoff of 0.05 were considered statistically significant for further analysis. The above indicated software modules were incorporated and executed in the Chipster v4 high-throughput data analysis platform (CSC)^78^. The commonly expressed upregulated and downregulated genes in lentiviral-SCR-treated versus lentiviral-shTie2-A-treated and lentiviral-SCR-treated versus lentiviral-shTie2-D-treated Tie2^+^ fibroblast cells with FDR < 0.05 were considered for gene ontology and pathway analysis using Metascape^79^.

### ELISA

To determine the secretion of mouse IL-6 and CCL5 in cultured Tie2^+^ fibroblasts, we grew the cells in 6-well plates in full RPMI 1640 medium described above until about 90% confluency. The cell cultures were transduced with two independent lentiviral clones, shTie2-A and shTie2-D, for 20 h. Lentiviral medium was then replaced with the full RPMI 1640 medium containing 1 µg ml^−1^ puromycin for 24 h, whereafter fresh full RPMI 1640 medium without a selection antibiotic was incubated for another 24 h, followed by change to serum-free RPMI 1640 medium that contained all of the other components. Aliquots of cell culture supernatants were collected after 3 h, 6 h, 12 h, 18 h and 24 h for determination of cytokines by commercial ELISA kits: DY478-05 (R&D Systems; mouse CCL5) and DY406-05 (R&D Systems; mouse IL-6).

### Immunohistochemistry and whole-mount immunostaining of tissue

Aortas were freshly embedded into Tissue-Tek O.C.T. Compound (Sakura Finetek USA), frozen in liquid nitrogen and stored at −80 °C. Ten micrometer sections were cut using cryotome (CryoStar NX70, Thermo Fisher Scientific) onto Superfrost plus glass (Thermo Fisher Scientific). Lung and liver were fixed in 4% paraformaldehyde (PFA) at 4 °C overnight, washed with PBS, followed by 30% sucrose for cryoprotection and embedded into Tissue-Tek O.C.T. Compound. Sections were dried at room temperature (20 °C) for 30 min and fixed with cold acetone for 10 min, then blocked in 2.5% donkey serum and 0.2% BSA in PBS for 1 h. The sections were then stained with primary antibodies in blocking solution overnight, followed by PBS washes and incubation with secondary antibodies.

For whole-mount immunostaining of intestine, PFA-fixed tissues were permeabilized in PBS containing 0.3% Triton X-100 at room temperature for 2 h and blocked in PBS containing 5% normal donkey serum, 0.2% BSA, 0.3% Triton X-100 and 0.05% NaN_3_ for 1 h. After blocking, tissues were stained with primary antibodies diluted in the blocking buffer at 4 °C for 2 days. After washes with PBS containing 0.3% Triton X-100 at room temperature, tissues were incubated overnight with fluorophore-conjugated secondary antibodies in PBS containing 0.3% Triton X-100 at 4 °C.

The following primary antibodies were used for immunostaining: goat anti-Tie2 (1:100), goat anti-VCAM1 (1:200), rat anti-mouse CD31 (1:500), rat anti-mouse CD68 (1:100), hamster anti-mouse CD3e (1:100), Cy3-conjugated anti-mouse SMA (1:500), goat anti-PECAM (1:300), rat anti-mouse Lyve1 (1:200) and sheep anti-mouse semaphorin 3C (1:100). The primary antibodies were detected with the appropriate Alexa Fluor 488 and Alexa Fluor 594 secondary antibody conjugates (diluted 1:500; Invitrogen). Supplementary Table 2 shows the list of antibodies used. Background autofluorescence was quenched using a TrueVIEW Autofluorescence Quenching Kit (Vector Laboratories) according to the manufacturer’s instructions. Stained samples were analyzed using confocal microscopy (Zeiss LSM 880 or Zeiss LSM 780). For chromogenic (peroxidase) staining, we used corresponding ImmPRESS kits from Vector Laboratories: Goat Anti-Rat IgG polymer kit (MP-7444) and Horse Anti-Goat IgG polymer kit (MP-7405). Images were generated using a 3DHISTECH PANNORAMIC 250 FLASH II digital slide scanner at the Genome Biology Unit supported by the Helsinki Institute of Life Science (HiLIFE) and the Faculty of Medicine, University of Helsinki, and Biocenter Finland.

### En face staining of aorta

*Tie2* was deleted by five consecutive daily intragastric administrations of tamoxifen to male mice. After the last day of tamoxifen treatment, the mice were allowed to rest in normal housing conditions for 2 days, then euthanized by anesthetic overdose. Aortas were quickly (2–3 min) isolated from mice, blood from the aortic lumen was flushed away with PBS and replaced with 4% PFA, and aortas were submerged in 4% PFA for 1 h fixation. The external part of the aortas was cleaned from other tissues and pinned to silicon plates for staining. Cleaned aortas were stained with Sudan IV and lipid area percentages were measured. Blocking and permeabilisation was done using donkey immunomix (BSA, 0.2%; normal donkey serum, 5%; sodium azide, 0.05%; Triton X-100, 0.3% in PBS). Primary antibodies (rabbit anti-Foxo1 (Cell Signaling Technology, 2880), 1:100) were added for 3 h, followed by a washing step (0.3% Triton X-100 in PBS) for 2 h with multiple changes of the wash buffer. Alexa Fluor 594 donkey anti-rabbit (Invitrogen; 1:500) was added for 2 h. After washing as above, Alexa Fluor 647 rabbit anti-ERG (Abcam, ab215228; 1:100) was incubated for 3 h for the staining of endothelial nuclei (indicated by the dashed line in the Fig. 7a). A final wash was 3 h long with multiple changes of the wash buffer, DAPI staining for nuclei (10 min) and post-fixation with 4% PFA for 10 min. For mounting, VECTASHIELD (Vector Laboratories, H-1000) was used.

### Real-time qPCR

For the mRNA analysis of the aorta, total RNA was isolated using the NucleoSpin RNA II Kit (Macherey-Nagel), and quality-controlled in a NanoDrop ND-1000 spectrophotometer. Reverse transcription to cDNA was performed using a High-Capacity cDNA Reverse Transcription Kit (Thermo Fisher Scientific). qPCR was carried out using a SensiFAST Probe No-ROX Kit (Bioline, BIO-86020) for TaqMan assay on a Bio-Rad C1000 thermal cycler. The TaqMan probes used were *Tie1* (Mm01180904), *Tie2* (Mm00443242) and *Gapdh* (4352932E). Gene expression was normalized to the *Gapdh* housekeeping gene, and fold changes were calculated using the comparative CT method.

### Statistical and reproducibility

All bar graphs show mean ± s.d. All animal experiments in the atherosclerosis model were performed twice with similar results. For comparisons of two experimental groups, two-tailed Student’s *t*-test was used (Graphpad Prism v9.0). In the analysis of the intravital microscopy experiments, we used two-way analysis of variance (ANOVA) with Sidak post hoc test. For CRISPRΔ, we used one-way ANOVA (*P* = 0.0024) with Dunnett’s post hoc test. For CCL5 and IL-6 ELISA, we used two-way ANOVA with Greenhouse–Geisser correction and Dunnet’s multiple comparisons test to the scramble control.

### Reporting summary

Further information on research design is available in the Nature Portfolio Reporting Summary linked to this article.

### Supplementary information

Supplementary Information Supplemental tables 1 and 2, and a figure with legend exemplifying the gating strategy.

Reporting Summary

Supplementary Data 1 Differentially expressed genes from clusters in Fig. 2b.

Supplementary Data 2 Differentially expressed genes from clusters in Extended Data Fig. 3d.

Supplementary Data 3 Differentially expressed genes from clusters in Extended Data Fig. 4g.

Supplementary Data 4 Ligand–receptor pairs from differentially expressed genes in *Tie2*^*iΔAEC*^ AECs in Fig. 2b.

Supplementary Data 5 Differentially expressed genes from AECs in Extended Data Fig. 5e.

Supplementary Data 6 Differentially expressed genes from Tie2^+^ fibroblasts in Fig. 5a.

Supplementary Data 7 Differentially expressed genes from AECs in Extended Data Fig. 9d.

### Source data

Source Data Fig. 1 Statistical source data for Fig. 1.

Source Data Fig. 2 Statistical source data for Fig. 2.

Source Data Fig. 3 Statistical source data for Fig. 3.

Source Data Fig. 4 Statistical source data for Fig. 4.

Source Data Fig. 5 Statistical source data for Fig. 5.

Source Data Fig. 6 Statistical source data for Fig. 6.

Source Data Fig. 7 Statistical source data for Fig. 7.

Source Data Extended Data Fig./Table 2 Statistical source data for Extended Data Fig. 2.

Source Data Extended Data Fig./Table 3 Statistical source data for Extended Data Fig. 3.

Source Data Extended Data Fig./Table 4 Statistical source data for Extended Data Fig. 4.

Source Data Extended Data Fig./Table 5 Statistical source data for Extended Data Fig. 5.

Source Data Extended Data Fig./Table 6 Statistical source data for Extended Data Fig. 6.

Source Data Extended Data Fig./Table 8 Statistical source data for Extended Data Fig. 8.

Source Data Extended Data Fig./Table 9 Statistical source data for Extended Data Fig. 9.

Source Data Extended Data Fig./Table 10 Statistical source data for Extended Data Fig. 10.

## Data Availability

All sequencing data are available in the GEO database (accession numbers GSE161979, GSE187844 and GSE187843). The CellChatDB.mouse ‘Secreated Signaling’ database is curated by the CellChat developer and loaded to R as instructed on CellChat.org. All other data supporting the finding in this study are included in the main article and associated files.
