# Supplementary material for: The angiopoietin receptor Tie2 is atheroprotective in arterial endothelium
Source: Nat Cardiovasc Res. 2023 Mar 13;2(3):307–21. doi: 10.1038/s44161-023-00224-y (PMC7614785; doi:10.1038/s44161-023-00224-y)
Supplement: Supplementary file 1 — Supplemental tables 1 and 2, and a figure with legend exemplifying the gating strategy. [file 44161_2023_224_MOESM1_ESM.pdf]

nature cardiovascular research

Article https://doi.org/10.1038/s44161-023-00224-y

The angiopoietin receptor Tie2 is atheroprotective in arterial endothelium

In the format provided by the authors and unedited

Supplementary Table 1:Primers used in the study			
**Gene**	**Primer ID**	**Sequence**	**Purpose**
Tie1	v3393	CATTGGACAGACAAGCATGG	Genotyping
	v3394	CCACCAGACAGAACCACTCA	Genotyping and recombination
	v3399	TAAAGGGTCGTCACGCTCCTAATTT	Recombination
Tie2(Tek)	TEKI3	CAGGCTATCACTGTGACACTGGTAC	Genotyping
	SDL2	AAATACGCAGTTTCAGGGCTGGGA	Genotyping and recombination
	VERI	ACCAATTCGGGGAATCCTATGGCA	Recombination
CRISPR deletion	L1:	GCAGGTTTAGTGAGCTCCAAGTTTTAGAGC TATGCT	Crispr gRNA
	R1:	GGAGTTGTAGCTTCCATACGGTTTTAGAGC TATGCT	Crispr gRNA
	R2:	AGTTGTACCTACAAGAAAGTGTTTTAGAGCTATGCT	Crispr gRNA
CRISPR-RNP deletion	Forward	CCTTTCCCTGTTGTGTGTTC	CRISPR-RNP deletion confirmation
	Reverse	CCGGCACAAGAATTACACTACC	CRISPR-RNP deletion confirmation
gRNA for rs1322052	gRNA	GTATGGACTTAGCAACAGAA	gRNA for rs1322052
TIE2(Tek)	FH1_TEK:	AAGACCTACGTGAATACCAC	gene expression confirmation
	BH1_TEK:	GAAACAGAGGGTATACAGATG	
RPLP0	[illegible]H1_RPLP0	AATCTCCAGGGGCACCATT	gene expression confirmation
	BH1_RPLP0	CGCTGGCTCCCACTTTGT	

Supplementary Table 2: Antibodies used in the study			
**Target protein**	**Host**	**Company**	**Cat. #**
VCAM1	Goat	R&D Systems	AF643
PECAM1	Rat	BD Bioscience	553370
TIE2 (TEK)	Goat	R&D Systems	AF762
CD68	Rat	Bio-Rad	MCA1957GA
CD3e	Hamster	BD Bioscience	553058
SEMAPHORIN 3C	Sheep	R&D Systems	AF1728
CD140a(PDGFRα)	Rat	Thermo Fisher Scientific	14-1401-81
Tie2-PE	Rat	Biolegned	124007
CD31-FITC	Rat	BD Bioscience	553370
PDGFRα-PE-Cy7	Rat	eBioscience	25-1401-81
CD45-Pacific Blue	Rat	Biolegned	103125
Ter119-Pacific Blue	Rat	Biolegned	116231
CD16/32 (Fc Block)	Rat	BD Bioscience	553141
B220-FITC	Rat	BD Bioscience	553088
CD3e-APC-eFluor780	Rat	eBioscience	47-0031
CD11b-PE-Cy7	Rat	eBioscience	25-0112
PE-CF594 anti mouse CD13	Rat	BD Bioscience	562537
Alexa 700 anti mouse CD127	Rat	eBioscience	56-1271-82
PerCP anti mouse CD45	Rat	BD Bioscience	557235
BV510 anti mouse CD11b	Rat	Biolegend	101263
PE anti mouse Gr1	Rat	eBioscience	12-5931-81
PE-Cy7 anti mouse F4/80	Rat	Biolegend	123114
APC anti mouse Fc Receptor	Rat	eBioscience	17-0161-81
eFluor450 anti mouse CD3e	Rat	eBioscience	48-0032
eFluor450 anti mouse B220	Rat	eBioscience	48-0452
eFluor450 anti mouse Gr1	Rat	eBioscience	48-5931
eFluor450 anti mouse CD11b	Rat	eBioscience	48-0112
PE anti mouse Sca-1	Rat	BD Bioscience	553108
PE-Cy7 anti mouse ckit	Rat	BD Bioscience	558163
Alexa Fluor 647 anti mouse CD34	Rat	BD Bioscience	560230
PE-CF594 anti mouse CD135	Rat	BD Bioscience	562537
Alexa 700 anti mouse CD127	Rat	eBioscience	56-1271-82

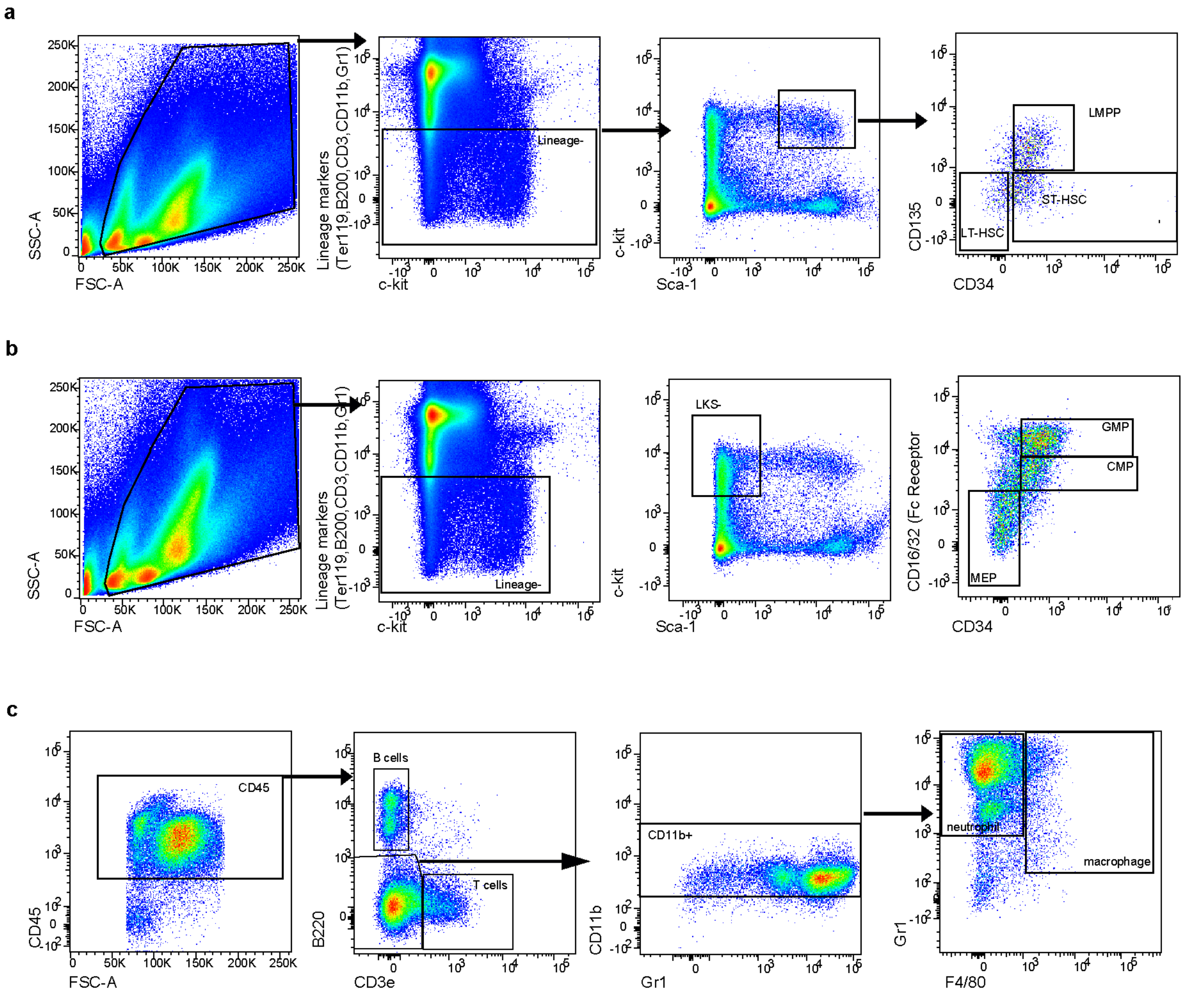

Supplementary Fig. 1 Gating strategy. a-c. Representative flow cytometry analysis with gating strategy on the hematopoietic stem and progenitor cells and mature immune cells.
