# Supplementary material for: The angiopoietin receptor Tie2 is atheroprotective in arterial endothelium
Source: Nat Cardiovasc Res. 2023 Mar 13;2(3):307–21. doi: 10.1038/s44161-023-00224-y (PMC7614785; doi:10.1038/s44161-023-00224-y)
Supplement: Supplementary file 2 — Reporting Summary [file 44161_2023_224_MOESM2_ESM.pdf]

nature portfolio

Corresponding author(s): Kari Alitalo

Last updated by author(s): Jan 23, 2023

Reporting Summary

Nature Portfolio wishes to improve the reproducibility of the work that we publish. This form provides structure for consistency and transparency in reporting. For further information on Nature Portfolio policies, see our Editorial Policies and the Editorial Policy Checklist.

Please do not complete any field with "not applicable" or n/a. Refer to the help text for what text to use if an item is not relevant to your study.
For final submission: please carefully check your responses for accuracy; you will not be able to make changes later.

Statistics

For all statistical analyses, confirm that the following items are present in the figure legend, table legend, main text, or Methods section.

n/a	Confirmed	
☐	☒	The exact sample size (*n*) for each experimental group/condition, given as a discrete number and unit of measurement
☐	☒	A statement on whether measurements were taken from distinct samples or whether the same sample was measured repeatedly
☐	☒	The statistical test(s) used AND whether they are one- or two-sided<br>*Only common tests should be described solely by name; describe more complex techniques in the Methods section.*
☒	☐	A description of all covariates tested
☐	☒	A description of any assumptions or corrections, such as tests of normality and adjustment for multiple comparisons
☐	☒	A full description of the statistical parameters including central tendency (e.g. means) or other basic estimates (e.g. regression coefficient) AND variation (e.g. standard deviation) or associated estimates of uncertainty (e.g. confidence intervals)
☐	☒	For null hypothesis testing, the test statistic (e.g. *F*, *t*, *r*) with confidence intervals, effect sizes, degrees of freedom and *P* value noted<br>*Give P values as exact values whenever suitable.*
☒	☐	For Bayesian analysis, information on the choice of priors and Markov chain Monte Carlo settings
☒	☐	For hierarchical and complex designs, identification of the appropriate level for tests and full reporting of outcomes
☒	☐	Estimates of effect sizes (e.g. Cohen's *d*, Pearson's *r*), indicating how they were calculated

Our web collection on statistics for biologists contains articles on many of the points above.

Software and code

Policy information about availability of computer code

Data collection

Images of Sudan IV-stained aortas were taken using digital camera Canon EOS 5D Mark IV, equipped with the objective Canon EF 100 mm f/2.8L Macro IS USM
Thin sections of aorta stained with the antibodies were imaged using an LSM880 confocal microscope (ZEISS). The carotid artery was exposed and the interaction of myeloid cells with the carotid artery was recorded using a 10x water-dipping lens.
En face aorta preparations stained with the antibodies were imaged using an LSM880 or LSM780 confocal microscopes (ZEISS).
Flow cytometry was carried out on the Becton Dickinson (BD) FACS Aria II Flow Cytometer/ BD FACSDiVa Software v 8.0
scRNAseq libraries were generated using the Chromium Single Cell 3' Library & Gel Bead Kit v2 or v3 (10X Genomics)
Total RNAseq libraries were generated using BGI DNBseq Stranded mRNA kit. 20 million paired end 100 (PE 100) reads were sequenced in the MGISEQ-2000 platform
qRT-PCR: Bio-Rad CFX Maestro™ Software v1.0
To measure the effect of CRISPR deletion and CRISPRi, RNA was reverse transcribed into cDNA using RevertAid First Strand cDNA Synthesis Kit (Themo Scientific) and qRT-PCR was performed.
For intravital microscopy, the carotid artery was exposed and the interaction of myeloid cells with the carotid artery was recorded using a 10x water-dipping lens.
For enzyme-Linked Immunosorbent Assay (ELISA) we used commercially available kits from R&D Systems, cat.# DY478-05 (mouse CCL-5) and cat.# DY406-05 (mouse IL-6).
To acquire publicly available data for GWAS and eQTL colocalization analysis we the CVD hugeamd portal (broadcvdi.org, 2021 11/30; http://cvd.hugeamp.org/region.html?chr=9&end=27280172&phenotype=CVD&start=27059139), converted to hg38 using USCS liftOver tool.

Data analysis

Z-stack and tile scan features were used to image the large aortic sections, stained with the antibodies. This was normally done using ZEN 2.0 Black software, ZEISS
Image J/FIJI (version 2.3.0/1.53q)
The digital expression matrix was generated by demultiplexing, barcode processing, and gene unique molecular index counting using the Cellranger count pipeline (version2.1.2, 10X Genomics).

HISAT2 (v2.2.1), HTseq (v0.13.5), DEseq2 (v3.16), Chipster (v4), Seurat (version 3.2.0), fGSEA (version 1.20.0), singleR (v1.8.1) and CellChat (version 1.4.0) was used to analyze the digital expression matrix.
Flow cytometry analysis was done using FlowJo v.10
The GWAS data was imported to ezQTL tool which performed colocalization analysis with GTEX v8 Tibial Artery data using HyPrColoc.
Statistical analyses were done using Excel (2016) or GraphPad Prizm v.9
Metascape, a gene annotation and analysis resource web tool, was used for gene ontology comparisons.
Metascape (https://metascape.org/)
FACSAria II flow cytometer with BD FACSDiVa Software v 8.0

For manuscripts utilizing custom algorithms or software that are central to the research but not yet described in published literature, software must be made available to editors and reviewers. We strongly encourage code deposition in a community repository (e.g. GitHub). See the Nature Portfolio guidelines for submitting code & software for further information.

Data

Policy information about availability of data

All manuscripts must include a data availability statement. This statement should provide the following information, where applicable:

- Accession codes, unique identifiers, or web links for publicly available datasets
- A description of any restrictions on data availability
- For clinical datasets or third party data, please ensure that the statement adheres to our policy

All sequencing data are available in the GEO database (accession number GSE161979, GSE187844, GSE187843). CellChatDB.mouse "Secreated Signaling" database are curated by CellChat developer and loaded to R as instructed on CellChat.org. All other data supporting the finding in this study are included in the main article and associated files.

Field-specific reporting

Please select the one below that is the best fit for your research. If you are not sure, read the appropriate sections before making your selection.

☒ Life sciences ☐ Behavioural & social sciences ☐ Ecological, evolutionary & environmental sciences

Life sciences study design

All studies must disclose on these points even when the disclosure is negative.

Sample size	Sample size for animal experiments was not specifically determined, and the n number in each experiment is indicated in figure legends. Sample size for each experiment is indicated in the legend. No statistical tests were used to pre-determine sample size, but sample size was chosen based on comparable studies in literature (Woo et al., J Clin Invest. 2011 Apr;121(4):1624-35).
Data exclusions	No data were excluded.
Replication	Data shown in figure panels are the mean of all independent biological repeats. Experimental findings were successfully replicated by authors.
Randomization	Because the experiments required a specific genetic signature, randomization per treatment was not possible. The animal studies were performed using littermate controls. For in vitro experiments no statistical methods were used for randomization. For scRNA-seq experiments, cells were isolated from randomly chosen wild-type or transgenic male mice.
Blinding	The investigators were blinded to group allocation during data collection and analysis.

Reporting for specific materials, systems and methods

We require information from authors about some types of materials, experimental systems and methods used in many studies. Here, indicate whether each material, system or method listed is relevant to your study. If you are not sure if a list item applies to your research, read the appropriate section before selecting a response.

Materials & experimental systems

n/a	Involved in the study	
	X	Antibodies
	X	Eukaryotic cell lines
X		Palaeontology and archaeology
	X	Animals and other organisms
X		Human research participants
X		Clinical data
X		Dual use research of concern

Methods

n/a	Involved in the study	
X		ChIP-seq
	X	Flow cytometry
X		MRI-based neuroimaging

Antibodies

Antibodies used

Antibodies used for immunohistochemistry:
VCAM1 Goat (1:200), R&D Systems, cat. # AF643
PECAM Goat (1:200), R&D Systems, cat. # AF3628
CD31 Rat (1:500), BD Pharmingen, cat. # 553370
Tie2 (TEK) Goat (1:100), R&D Systems, cat. # AF762
CD68 Rat (1:100), Bio-Rad, cat. # MCA1957GA
CD3e Hamster (1:100), BD Pharmingen, cat. # 553058
Semaphorin 3C Sheep (1:100), R&D Systems, cat. # AF1728
Lyve-1 Rat (1:200), R&D Systems, cat. # MAB2125
SMA-Cy3 Mouse (1:500), SigmaAldrich, cat. # C6198

Secondary antibodies, conjugated to fluorophores:
Donkey anti-mouse-Alexa488 (1:500), Invitrogen, cat. # A21202
Donkey anti-goat-Alexa488 (1:500), Invitrogen, cat. # A11055
Donkey anti-rat-Alexa488 (1:500), Invitrogen, cat. # A21208
Donkey anti-rabbit-Alexa488 (1:500), Invitrogen, cat. # A21206
Donkey anti-goat-Alexa594 (1:500), Invitrogen, cat. # A11058
Donkey anti-rabbit-Alexa594 (1:500), Invitrogen, cat. # A21207
Donkey anti-rat-Alexa594 (1:500), Invitrogen, cat. # A21209
Donkey anti-mouse-Alexa594 (1:500), Invitrogen, cat. # A21203
Donkey anti-sheep-Alexa594 (1:500), Invitrogen, cat. # A11016

Antibodies used for FACS sorting and analysis:
Tie2-PE Rat (1:100), Biolegends, cat. # 124007
CD31-FITC Rat (1:100), BD Pharmingen, cat. # 553370
PDGFRα-PE-Cy7 (1:100), eBioscience, cat. # 25-1401-81
CD45-Pacific Blue Rat (1:100), Biolegend, cat. # 103125
Ter119-Pacific Blue Rat (1:100), Biolegend, cat. # 116231
CD16/32 (Fc Block) Rat (1:100), BD Pharmingen, cat. # 553141
FITC anti mouse B220 (1:100,Clone RA3-6B2, BD Pharmingen, 553088)
APC-eFluor780 anti mouse CD3e (1:100,Clone 145-2C11, eBioscience, 47-0031)
PE-Cy7 anti mouse CD11b (1:100,Clone M1/70, eBioscience, 25-0112)
PE-CF594 anti mouse CD135 (1:100, Clone A2F10.1, BD Pharmingen, 562537)
Alexa 700 anti mouse CD127 (1:100, Clone A7R34, eBioscience, 56-1271-82)
PerCP anti mouse CD45 (1:100, 30-F11, BD Pharmingen,557235)
BV510 anti mouse CD11b (1:100, Clone M1/70, Biolegend, 101263)
PE anti mouse Gr1 (1:100,Clone RB6-8C5, eBioscience, 12-5931-81)
PE-Cy7 anti mouse F4/80 (1:100, Clone BM8, Biolegend, 123114)
APC anti mouse Fc Receptor (1:200, clone 93, eBioscience, 17-0161-81)
eFluor450 anti mouse CD3e (1:100,Clone 17A2, eBioscience, 48-0032)
eFluor450 anti mouse B220 (1:100,Clone RA3-6B2, eBioscience, 48-0452)
eFluor450 anti mouse Gr1 (1:100,Clone RB6-8C5, eBioscience, 48-5931)
eFluor450 anti mouse CD11b (1:100,Clone M1/70, eBioscience, 48-0112)
PE anti mouse Sca-1 (1:100,Clone D7, BD Parmingen, 553108)
PE-Cy7 anti mouse ckit (1:100, Clone 2B8, BD Parmingen, 558163)
Alexa Fluor 647 anti mouse CD34 (1:100, Clone RAM34, BD Parmingen, 560230)

Validation

All antibodies were obtained from indicated commercial vendors with ensured quality and validated by the commercial vendors for specificity in the indicated species and application. The specificity of staining was controlled based on simultaneous analysis of cell populations known to lack expression of the relevant antigens. All experiments included single staining controls and, where possible, different fluorescence-minus-one (FMO) controls and staining panels included internal controls (known negative and positive populations) to validate specific antibody signals.

Eukaryotic cell lines

Policy information about cell lines

Cell line source(s)	HEK293 human embryonic kidney cells, ATCC, cat. # CRL-3216<br>TeloHAEC (ATCC, CRL-4052)
Authentication	Authentication was guaranteed by the vendor. Morphology is used for HEEK293. Morphology and puromycin selection are used to authenticate teloHAECs.
Mycoplasma contamination	Mycoplasma levels were routinely tested in 293T cells and were mycoplasma free. teloHAECs are not tested for mycoplasma contamination.
Commonly misidentified lines (See ICLAC register)	None.

Animals and other organisms

Policy information about studies involving animals; ARRIVE guidelines recommended for reporting animal research

Laboratory animals	All mouse strains (The Tie1fl/fl, Tie2fl/fl , BmxCreERT, and BmxLacZ) mouse strains were maintained on a C57BL/6J background. All mouse strains used are reported in Methods: "Mice and Tissues". Only adult male mice (10-12 weeks) were used in our experiments. Mice were provided with water and food ad libitum and on a 12-h light-dark cycle and kept at 22± 2 °C with relative humidity of 55 ± 10%.
Wild animals	No wild animals were used in this study
Field-collected samples	No field-collected samples were used in this study
Ethics oversight	All animal experiments were approved by the Committee for Animal Experiments of the District of Southern Finland.

Note that full information on the approval of the study protocol must also be provided in the manuscript.

Flow Cytometry

Plots

Confirm that:

☒ The axis labels state the marker and fluorochrome used (e.g. CD4-FITC).

☒ The axis scales are clearly visible. Include numbers along axes only for bottom left plot of group (a 'group' is an analysis of identical markers).

☒ All plots are contour plots with outliers or pseudocolor plots.

☒ A numerical value for number of cells or percentage (with statistics) is provided.

Methodology

Sample preparation	Aortic cells, bone marrow and peripheral blood from wildtype and transgenic mice were prepared into single cell suspension in HBSS supplemented with fetal calf serum. All samples were incubated with Fc-Block prior to staining with monoclonal antibodies.
Instrument	Isolated cells were stained with fluorochrome-conjugated antibody and analyzed or sorted by FACSAria IIu (Becton Dickinson)
Software	Data were collected using BD FACSDiVa Software v 8.0 and analyzed using FlowJo software (Tree Star) v10
Cell population abundance	Post-sort purity analysis is not possible for single cell sort but several measures were done to ensure purity. First, a test sort and reanalysis of a relevant cell population prior to performing 96-well cell sort was done. Typically 100-1000 cells were first sorted and purity upon reanalysis was >95% percent when factoring in all impurities in all hierarchical gates. Secondly, accurate single cell deposition was validated using fluorescent beads.
Gating strategy	Cells were gated on singlets (standard gating strategy), forward/side scatter, and antibody stating. DAPI-negative cells were gated out to exclude non-viable cells where possible.

☒ Tick this box to confirm that a figure exemplifying the gating strategy is provided in the Supplementary Information.

This checklist template is licensed under a Creative Commons Attribution 4.0 International License, which permits use, sharing, adaptation, distribution and reproduction in any medium or format, as long as you give appropriate credit to the original author(s) and the source, provide a link to the Creative Commons license, and indicate if changes were made. The images or other third party material in this article are included in the article's Creative Commons license, unless indicated otherwise in a credit line to the material. If material is not included in the article's Creative Commons license and your intended use is not permitted by statutory regulation or exceeds the permitted use, you will need to obtain permission directly from the copyright holder. To view a copy of this license, visit http://creativecommons.org/licenses/by/4.0/
